# Unveiling the Ionic Diels–Alder Reactions within the Molecular Electron Density Theory

**DOI:** 10.3390/molecules26123638

**Published:** 2021-06-14

**Authors:** Luis R. Domingo, Mar Ríos-Gutiérrez, María José Aurell

**Affiliations:** Department of Organic Chemistry, University of Valencia, Dr. Moliner 50, E-46100 Valencia, Spain; M.Mar.Rios@uv.es (M.R.-G.); maria.j.aurell@uv.es (M.J.A.)

**Keywords:** ionic Diels–Alder reactions, Molecular Electron Density Theory, iminium cations, superelectrophiles, global electron density transfer, asynchronicity

## Abstract

The ionic Diels–Alder (I-DA) reactions of a series of six iminium cations with cyclopentadiene have been studied within the Molecular Electron Density Theory (MEDT). The superelectrophilic character of iminium cations, ω > 8.20 eV, accounts for the high reactivity of these species participating in I-DA reactions. The activation energies are found to be between 13 and 20 kcal·mol^−1^ lower in energy than those associated with the corresponding Diels–Alder (DA) reactions of neutral imines. These reactions are low *endo* selective as a consequence of the cationic character of the TSs, but highly regioselective. Solvents have poor effects on the relative energies, and an unappreciable effect on the geometries. In acetonitrile, the activation energies increase slightly as a consequence of the better solvation of the iminium cations than the cationic TSs. Electron localization function (ELF) topological analysis of the bonding changes along the I-DA reactions shows that they are very similar to those in polar DA reactions. The present MEDT study establishes that the global electron density transfer (GEDT) taking place at the TSs of I-DA reactions, and not steric (Pauli) repulsions such as have been recently proposed, are responsible for the features of these types of DA reactions.

## 1. Introduction

**Scheme 1:**
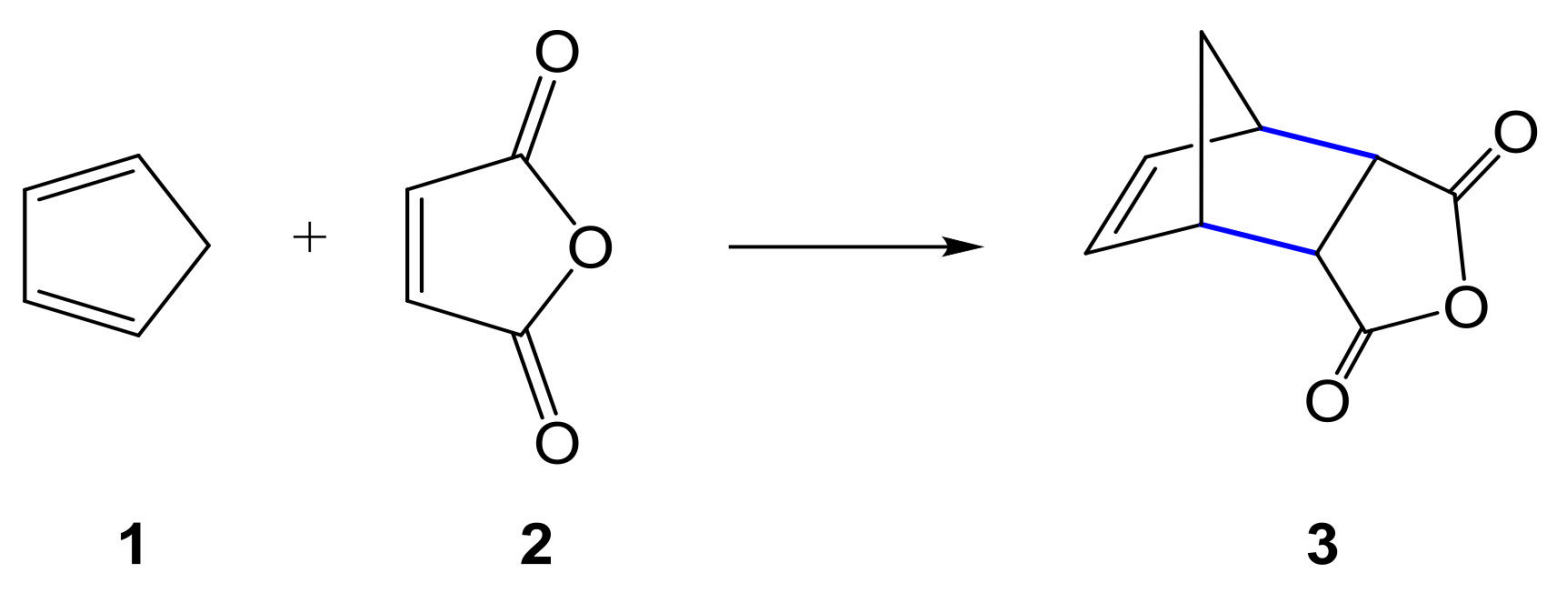
DA reaction between Cp **1** and maleic anhydride **2** reported in 1928 by Diels and Alder.

The Diels–Alder (DA) reaction between a conjugated diene and an ethylene to yield a cyclohexene, reported for the first time by Diels and Alder in 1928 [1], is one of the most studied organic reactions from a synthetic as well as a theoretical viewpoint (see Scheme 1) [2,3].

**Scheme 2:**
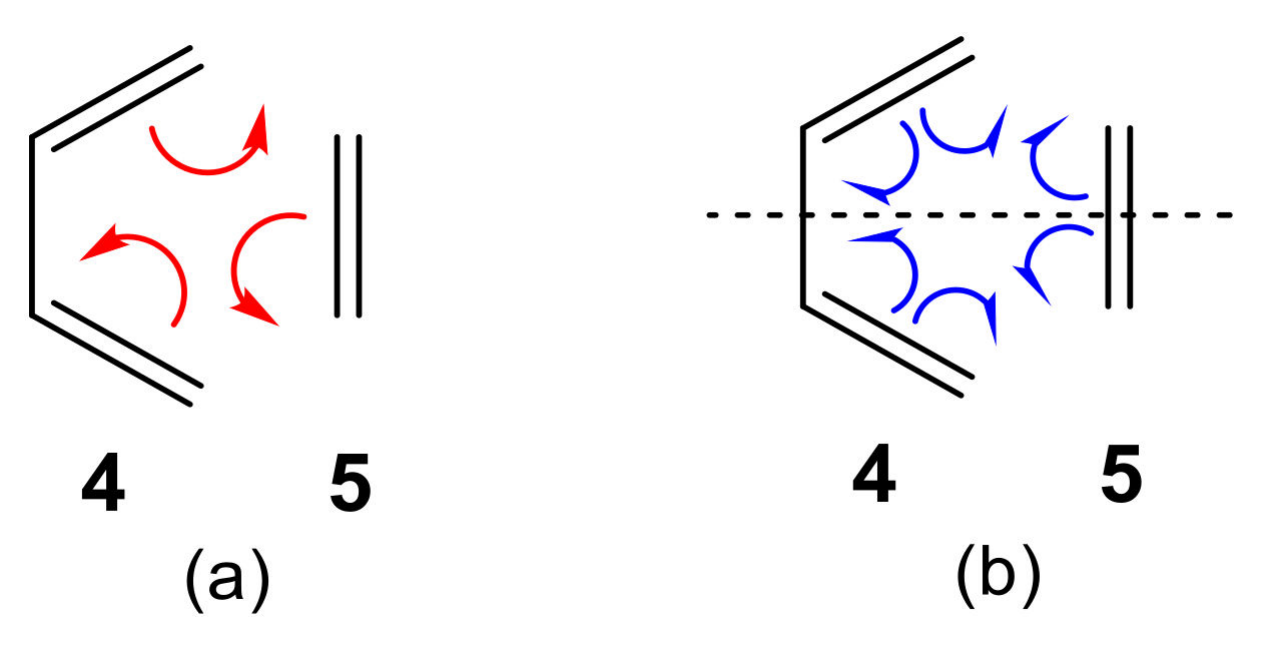
Changes in electron density in the DA reaction between butadiene **4** and ethylene **5**: (**a**) those proposed in the pericyclic mechanism [4]; (**b**) those resulting from a BET analysis [8].

The mechanism of the DA reaction has been the subject of many theoretical studies. The most accepted one is the “pericyclic mechanism” proposed by Woodward and Hoffman in 1969 [4], and further supported by Houk in 1995 [5]. The “pericyclic mechanism” concept refers as the electron density changes along the DA reactions, but this mechanism was not quantum chemically proved until 2002, when a bonding evolution theory [6] (BET) study of the DA reaction between butadiene **4** and ethylene **5** showed that the changes in electron density take place sequentially along a symmetrical plane (see Scheme 2) [7,8].

After an exhaustive study of organic reactions carried out over 20 years, in 2016, Domingo proposed the Molecular Electron Density Theory [9] (MEDT) for the study of the organic reactivity. MEDT contrasts against any reactivity model based on the analysis of molecule orbitals (MOs) such as the proposed frontier molecular orbital (FMO) theory [10], enabled rejections of both the “pericyclic mechanism” [8] proposed by Woodward and Hoffman and the “aromatic transition state structures (TSs)” proposed by Dewar [11].

**Figure 1:**
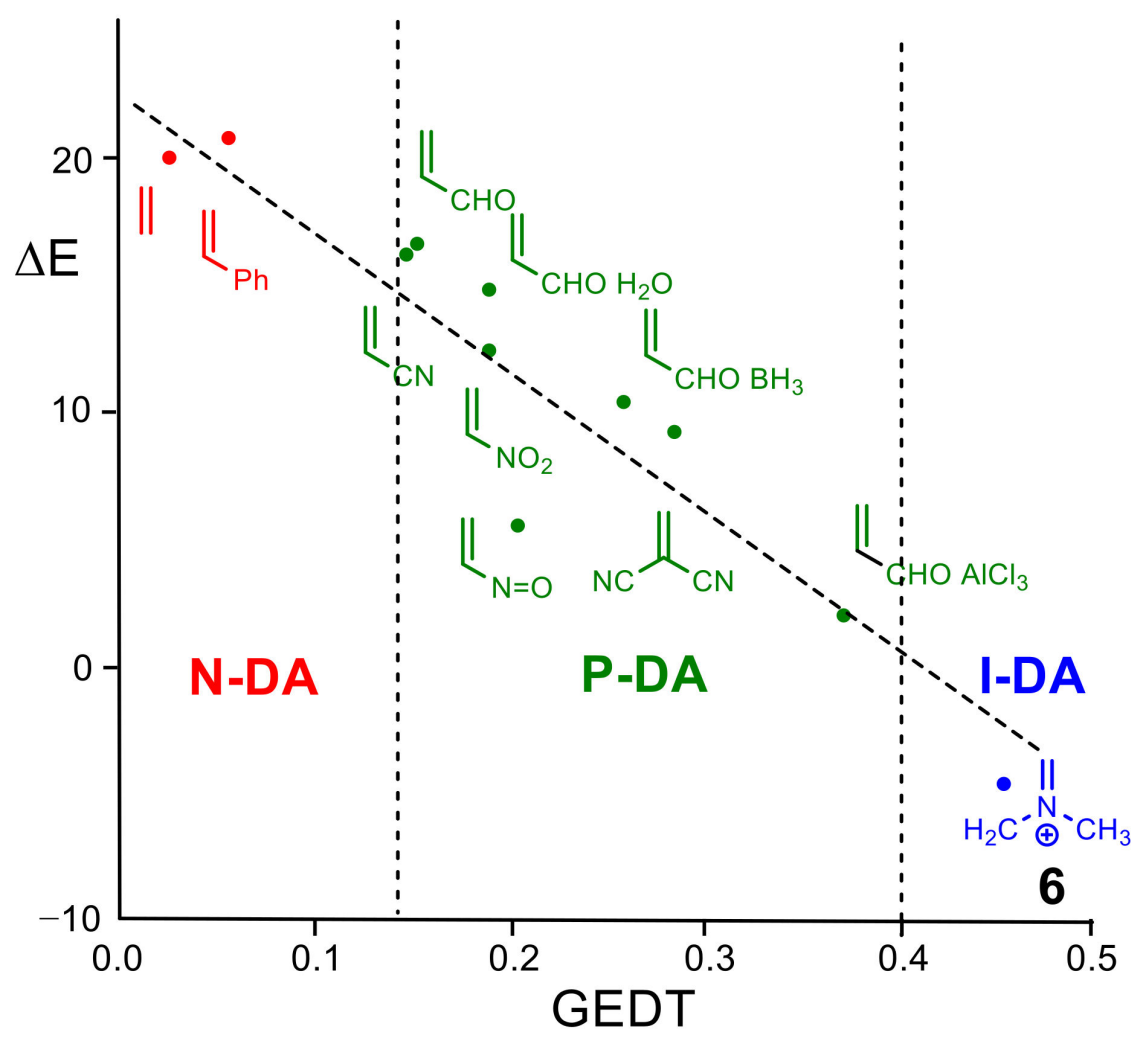
Plot of the activation barriers (ΔE in kcal·mol^−1^) vs. the GEDT, e, *R*^2^ = 0.89, for the DA reactions of Cp **1** with the substituted ethylene series of an increased electrophilic character. The classification of the DA reactions, based on the GEDT, is included.

An exhaustive theoretical study on experimental DA reactions established the definitive role of the global electron density transfer [12] (GEDT) taking place at the TSs in the feasibility of DA reactions [13,14]. In 2009, this finding established the mechanism of polar DA (P-DA) reactions [15], in which the favorable nucleophilic/electrophilic interactions taking place at the TSs are responsible for the feasibility of the reaction. The very good correlation found between the GEDT and the activation barriers for the DA reactions of Cp **1** with a series of 12 substituted ethylenes, including iminium cation **6**, enabled the classification of the DA reactions in non-polar DA (N-DA), which do not take place easily experimentally, P-DA reactions, and ionic DA (I-DA) reactions, in which one of the two reagents is an ionic species (see Figure 1) [15]. In this sense, analysis of the electrophilicity [16] ω and nucleophilicity [17] *N* indices, defined within the conceptual DFT [18,19] (CDFT), has becomes a powerful tool for experimentalist organic chemists to easily study DA reactions [15].

Iminium cation **6** was found to be the most reactive species, showing the highest electrophilicity ω index, 8.24 eV. This very high value, which classifies it as a superelectrophile [20,21], is a consequence of its cationic nature. Although I-DA reactions can be understood as the extreme of P-DA reactions, the different behaviors of the TSs involved in I-DA reactions, with respect to those of P-DA reactions, demanded their classification as I-DA reactions (see Figure 1) [15].

**Scheme 3:**
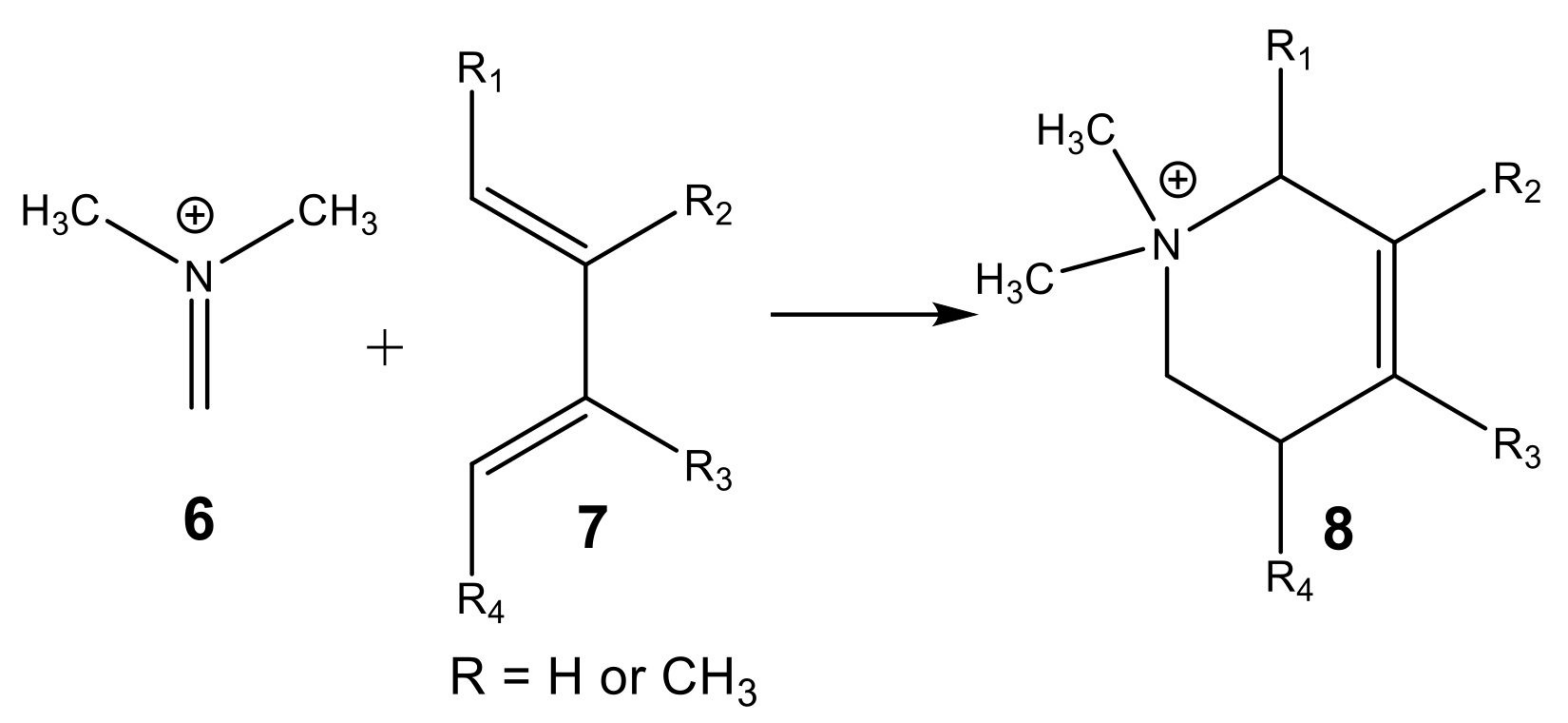
I-DA reactions experimentally and theoretically studied by Sauer et al. [22].

Many I-DA reactions have been described in the literature; however, the majority of them involves cationic species such as cationic derivatives of imines. This behavior is a consequence of the fact that whereas superelectrophilic species react with poor or marginal nucleophiles, supernucleophilic species, such as anionic species, do no react effectively with poor or marginal electrophiles. Thus, Sauer et al. studied the DA reactions between several salts of iminium cation **6** and substituted butadienes **7**, both experimentally and theoretically using AM1 semiempirical calculations (see Scheme 3) [22]. These authors suggested that the DA reactions of iminium cation **6** with the acyclic 1,3-butadienes **7** proceeded via transition state structures (TSs) that closely resemble those yielding intermediate allyl cations. Only in the case of reaction with Cp **1** did these authors conclude that the reaction takes place along a pericyclic TS [22].

**Scheme 4:**
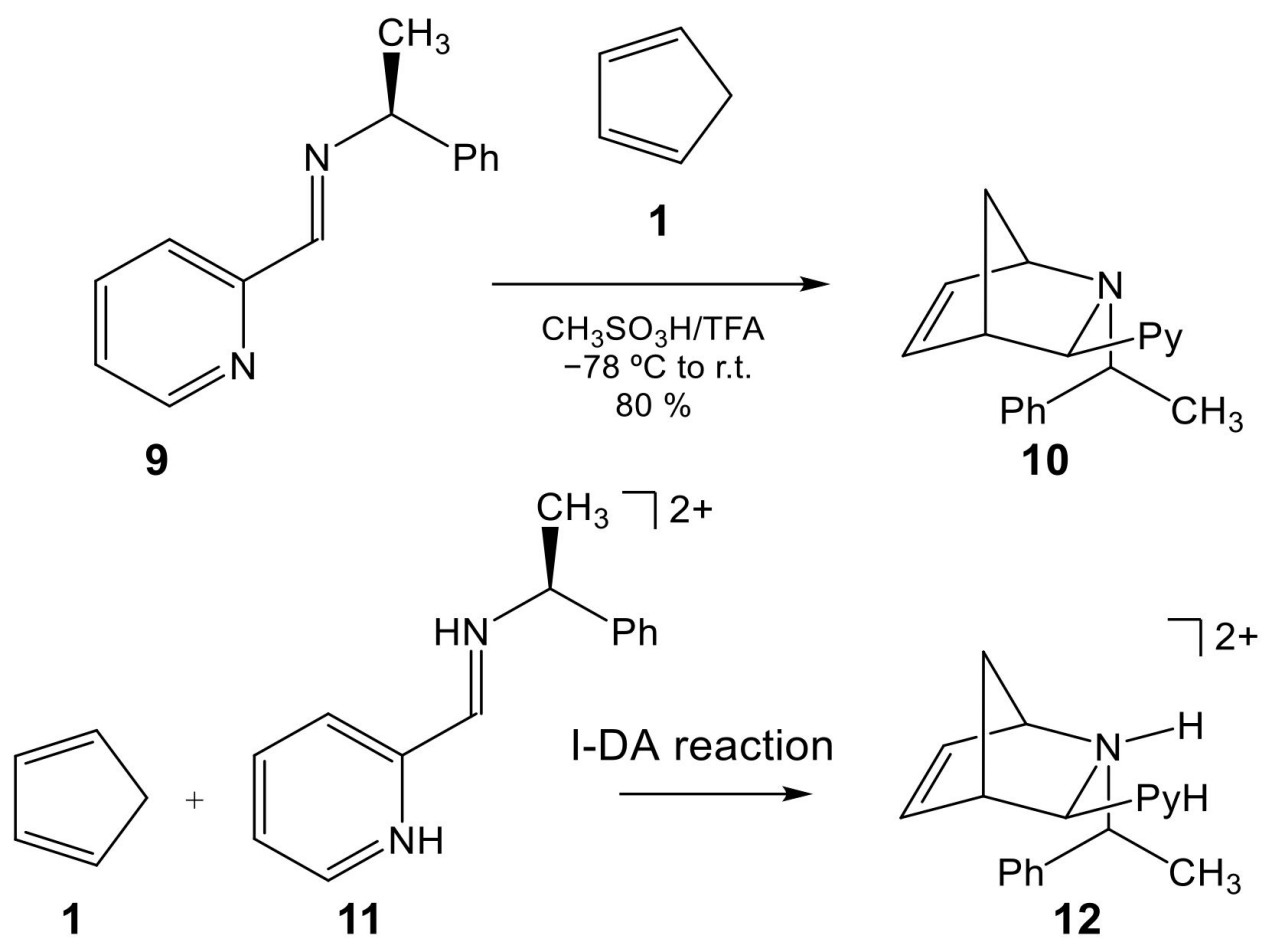
I-DA reactions experimentally studied by Andersson et al. [23].

Andersson et al. explored the DA reaction between Cp **1** and a family of chiral imines containing a second nitrogen atom at a conjugated position, because in a strong acid medium, the protonation of this nitrogen atom might fulfil the role of an electron-withdrawing group [23]. Thus, when imine **9** was treated with Cp **1** under strong acid conditions at −78 °C, a highly stereoselective cycloaddition took place, giving the pyridine-substituted aza-norbornene **10** in good yield (see Scheme 4). Note that this reaction is a Bronsted acid-catalyzed DA reaction of the imine **9**.

The participation of cationic species as the heterodiene or the ethylene derivative in DA reactions has been widely studied in the literature. Thus, in 2001, the I-DA reaction between iminium cation **6** and Cp **1**, experimentally studied by Sauer [22], was studied at the B3LYP/6-31G(d) and MP2/6-31G(d) levels [24]. This study demonstrated that this I-DA reaction takes place via a one-step mechanism involving a highly asynchronous TS with a high GEDT. A complete analysis of the features of the TS indicated that this I-DA reaction can be characterized by the nucleophilic attack of Cp **1** on the carbon of the electron-poor iminium cation **6** instead of a “pericyclic process”. The concomitant ring closure affords the final cycloadduct without the participation of any allyl cation intermediate, via a non-concerted *two-stage one-step* mechanism [25]. Consequently, iminium cation **6** and Cp **1** behave as an electrophile and nucleophile, respectively, instead of as dienophile and diene.

The I-DA reaction of Cp **1** with diprotonated imine **11**, experimentally reported by Andersson [23], was theoretically studied using HF/6-31G(d) and B3LYP/6-31G(d) methods [26]. This I-DA reaction takes place along a two-step mechanism. The first step corresponds to the nucleophilic attack of Cp **1** on the carbon of the iminium cation group to produce an acyclic cation intermediate, while the second step is associated with the ring closure of this intermediate along the formation of the second C–N single bond, yielding the final cycloadduct **12**. Protonation of both pyridine and imine nitrogen atoms strongly increases the electrophilicity of the imine, and the I-DA reaction takes place along a favorable inverted energy profile, allowing the justification of the large acceleration of the reaction in a strong acid medium [23]. The inclusion of solvent effects yielded a negligible variation of the energy profile.

In 2014, Domingo performed a quantum chemical topological analysis of the C–C bond formation in organic reactions involving cationic species [27]. To this end, the I-DA reaction between the iminium cation **6** and Cp **1** was chosen as a computational model. The BET analysis showed that C–C bond formation begins at the C–C distance of 1.96 Å via a C-to-C *pseudoradical* coupling between the most electrophilic center of the iminium cation and one of the two most nucleophilic centers of Cp. Interestingly, this pattern for the formation of the C–C single bond was identical to that found in the N-DA reaction between butadiene **4** and ethylene **5** [12]. Analysis of the Parr functions [28] enabled characterization of the most electrophilic and nucleophilic centers of the reagents participating in this I-DA reaction.

Very recently, Bickelhaup et al. studied the origin of rate enhancement and asynchronicity in iminium-catalyzed DA reactions using an energy decomposition analysis [29]. Through their activation strain model (ASM) [30] and Kohn–Sham molecular orbital [31] analyses, these authors suggested that “the iminium catalysts enhance the reactivity by reducing the steric (Pauli) repulsion between the diene and dienophile, which originates from both a more asynchronous reaction mode and a more significant polarization of the π-system away from the incoming diene compared to aldehyde and imine analogs”. They concluded that “the driving force behind the asynchronicity of these DA reactions is the relief of destabilizing steric (Pauli) repulsion and not the orbital interaction between the terminal carbon of the dienophile and the diene” [29].

**Figure 2:**
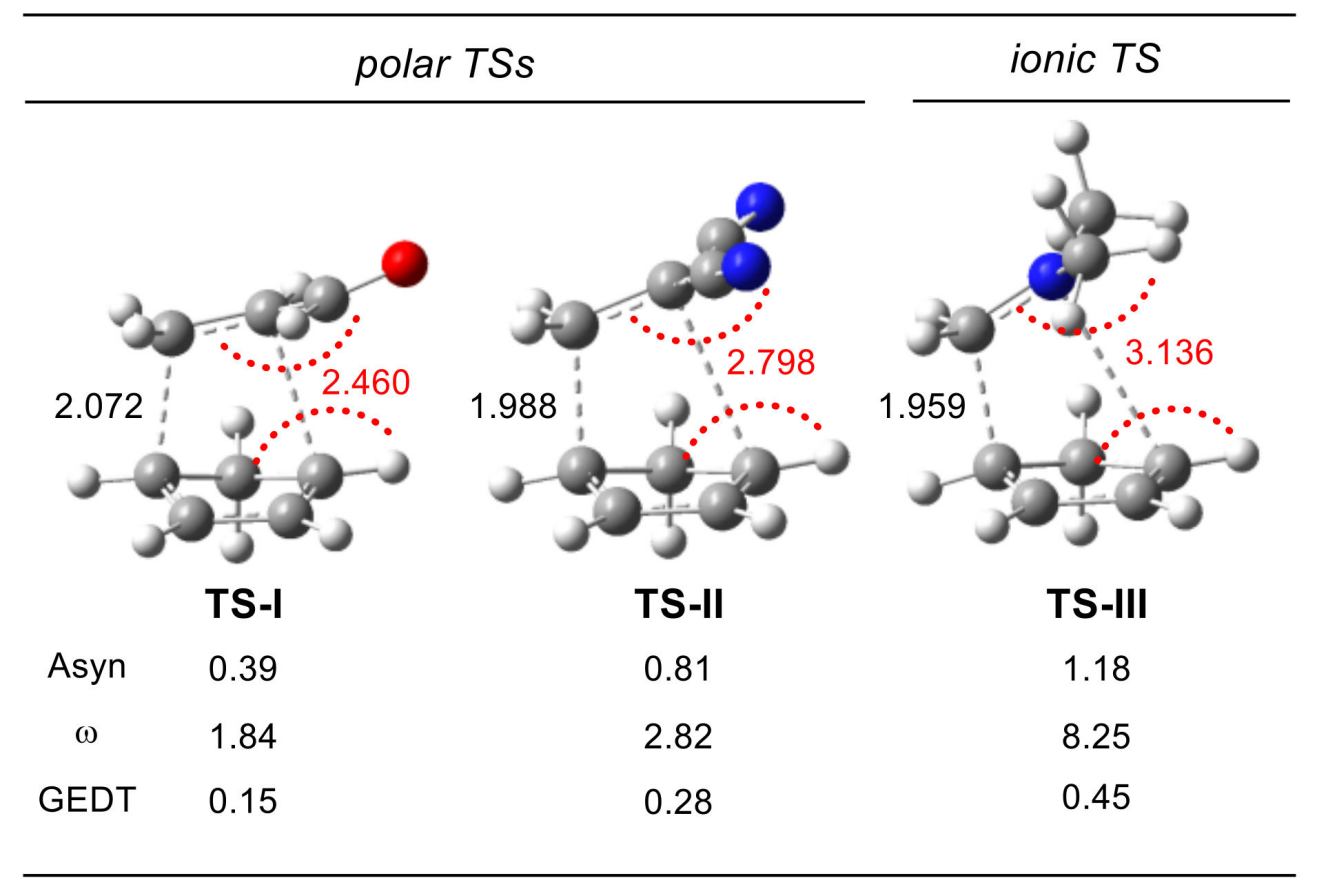
B3LYP/6-32G(d) Asynchronicity, Asyn = d1-d2 in Angstroms, Å, electrophilicity ω index of the ethylene, in eV, and GEDT, in e, at the TSs involved in P-DA reactions, **TS-I** and **TS-II**, and in I-DA reaction, **TS-III**.

The asynchronicity in cycloaddition reactions has been widely studied within the polar reaction model [13,14,15]. P-DA reactions involving symmetric electrophilic ethylenes such as anhydride maleic or tetracyanoethylene take place through synchronous TSs [14], whereas the use of non-symmetric electrophilic ethylenes such as nitroethylene or 1,1-dicyanoethylene yields highly asynchronous TSs [13,14,15,32] These studies emphasized that along an asynchronous single bond formation process, the more favorable two-center interaction begins at the most electrophilic and nucleophilic centers of the two interacting molecules. A good linear correlation between the GEDT and the asynchronicity at eight of the TSs associated with the P-DA and I-DA reactions given in Figure 1 can be established, *R*^2^ = 0.95 (see Figure S1 in Supplementary Material). As shown, the asynchronicity increases with the increase in the polar character of the reaction, i.e., the increase in the GEDT at the TSs [13,14,15,32]. The stronger the nucleophilic/electrophilic interactions at the TSs involving non-symmetric electrophiles, the higher the asynchronicity. As a consequence, I-DA reactions can take place through a non-concerted *two-stage one-step* mechanism [25] via a highly asymmetric TS [24], even though a two-step mechanism involves a cation intermediate [26]. The increase in the asynchronicity enhances the distance between the ends of the two interacting frameworks, the iminium cation, and the diene (see **TS-III** in Figure 2), and consequently, decreases the steric (Pauli) repulsions. Consequently, the decrease in steric (Pauli) repulsions is a consequence of the increase in the asynchronicity, and not vice versa; asynchronicity in P-DA and I-DA reactions depends on the GEDT taking place at the TSs, which depends on the electrophilic/nucleophilic nature of the reagents.

**Scheme 5:**
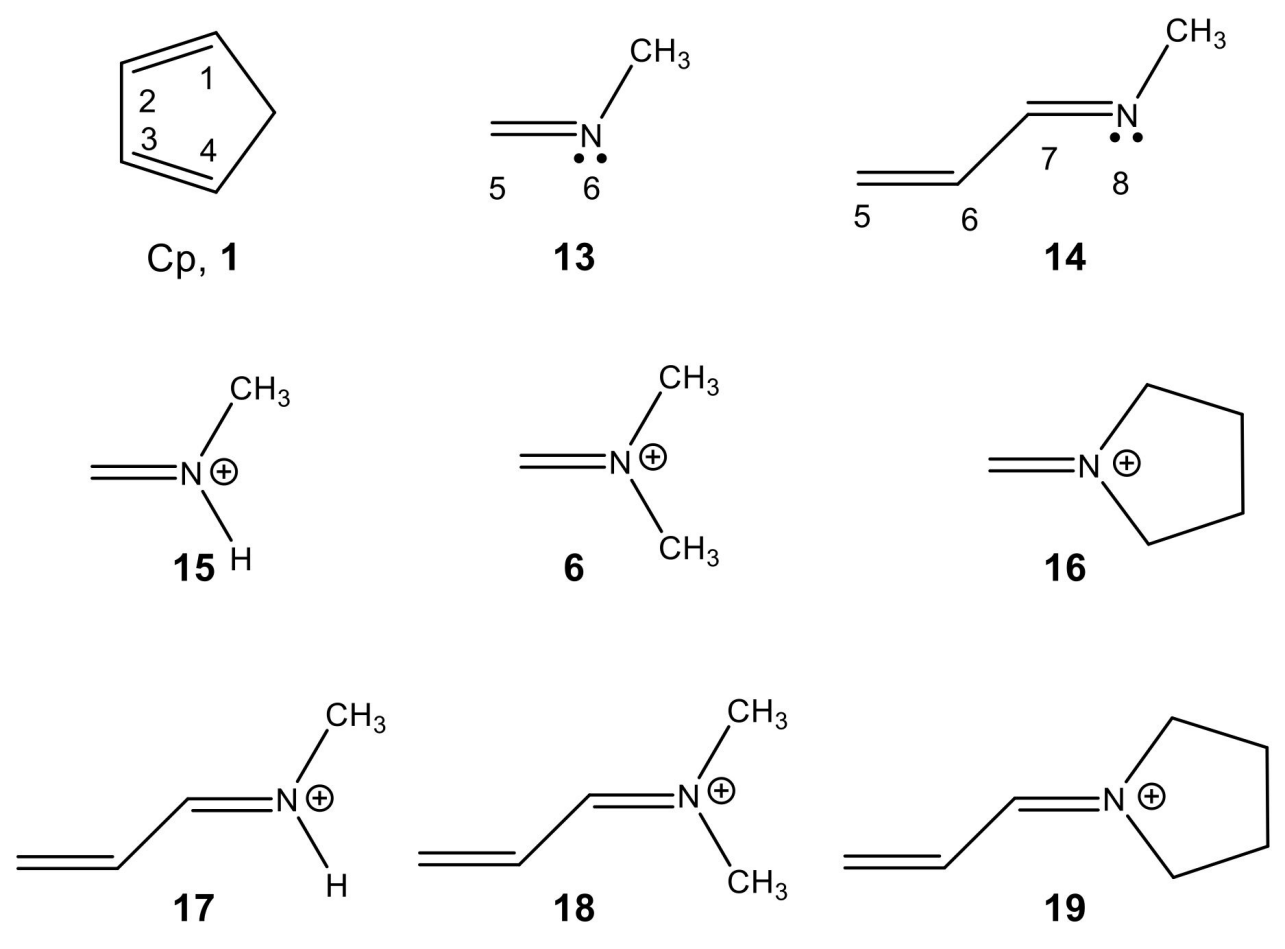
Structures of the species involved in the P-D reactions of Cp **1** with imines **13** and **14**, and the I-DA reactions with iminium cation **6**, **15**–**19**.

Although I-DA reaction can be viewed as the extreme of P-DA reactions [15], they have many behaviors different from P-DA reactions, i.e., I-DA reactions are poorly *endo* selective, and polar solvents cause a contrary effect in P-DA reactions. Herein, a complete MEDT study of I-DA reactions involving cationic species is presented. To this end, the I-DA reactions of six iminium cations, **6**, **15**–**19**, with Cp **1** were selected as computational models (see Scheme 5). It is interesting to remark that protonated imines **15** and **17** can be considered as species participating in acid Bronsted-catalyzed DA reactions of imines **13** and **14** [23].

The present MEDT study responds to the following questions: (i) What is the origin of the high acceleration and asynchronicity found in I-DA reactions?; (ii) What is the origin of the low *endo* selectivity in contrast with the high *endo* selectivity found in P-DA reactions?; and (iii) What is the role of the solvent polarity in these I-PA reactions?

## 2. Results and Discussions

The present MEDT study has been divided into three parts: (i) first, a study of the electronic structure and reactivity at the ground state (GS) of the reagents is performed; (ii) in the second part, a study of the potential energy surface of the I-DA reactions of iminium cations **6**, **15**–**19** with Cp **1** is carried out; and finally, (iii) in the third part, a topological analysis of the bonding changes along the I-DA reactions is presented. The origin of the regioselectivity in I-DA reactions and asynchronicity in bond formation at the TSs is analyzed on the basis of changes in electron density.

### 2.1. Study of the Electronic Structure and Reactivity at the Ground State of the Reagents

#### 2.1.1. Analysis of the Electronic Structure of Imines and Iminium Cations

**Figure 3:**
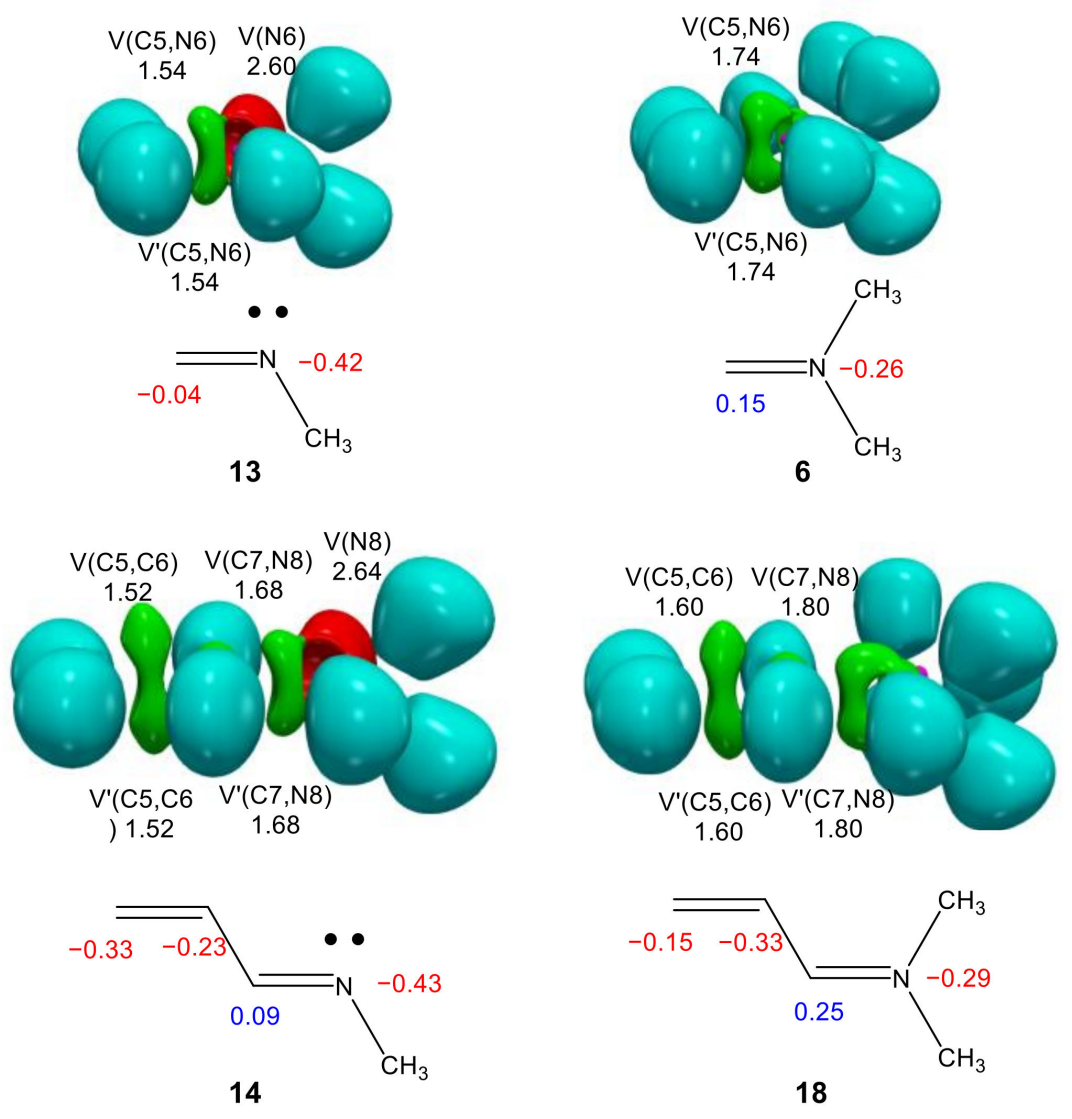
ωB97XD/6-311G(d,p) localization domains (isosurface value = 0.75): blue color is used to show protonated basins, red color for the monosynaptic basins, green color for the disynaptic basins and magenta color for core basins, ELF based Lewis-like structures, and natural atomic charges, in average number of electrons e, blue color is used for positive charges and red color for negative charges, of imines **13** and **14**, and iminium cations **6** and **18**.

In order to obtain information about how the formation of the iminium cations modifies the electronic structure of imines, electron localization function [33] (ELF) and natural population analysis [34,35] (NPA) assays of imines **13** and **14**, and iminium cations **6** and **18**, was performed. The ELF valence basin localization domains, the ELF-based Lewis-like structures, and natural atomic charges are presented in Figure 3.

ELF of imine **13** shows the presence of two disynaptic basins, V(C5,N6) and V′(C5,N6), integrating a total of 3.08 e, one V(N6) monosynaptic basin integrating 2.60 e, and one V(N6,C(Me)) disynaptic basin, integrating 1.74 e. The two disynaptic basins are associated with the underpopulated C5=N6 double bond, and the monosynaptic basin is associated with the non-bonding electron density of the N6 nitrogen. ELF of conjugated imine **14** shows the presence of two disynaptic basins, V(C5,C6) and V′(C5,C6), integrating a total of 3.04 e, one V(C6,C7) disynaptic basins integrating 2.22 e, two disynaptic basins, V(C7,N8) and V′(C7,N8), integrating a total of 3.36 e, and one V(N8) monosynaptic basin, integrating 2.64 e. The two V(C5,C6) and V′(C5,C6) disynaptic basins are associated with the underpopulated C5=C6 double bond, the V(C6,C7) disynaptic basin is associated with the overpopulated C6–C7 single bond, the V(C7,N8) and V′(C7,N8) disynaptic basins are associated with the underpopulated C7=N8 double bond, and the monosynaptic basin is associated with the non-bonding electron density of the N8 nitrogen. In addition, one V(N8,C(Me)) disynaptic basin, integrating 1.75 e, associated with the N8-C(Me) single bond is observed.

ELF of iminium cation **6** shows the presence of two disynaptic basins, V(C5,N6) and V′(C5,N6), integrating a total of 3.48 e, and two disynaptic basins, V(N6, C(Me)) and V(N6, C′(Me)), integrating 1.92 e each. ELF of conjugated iminium cation **18** shows the presence of two disynaptic basins, V(C5,C6) and V′(C5,C6) integrating a total of 3.20 e, one V(C6,C7) disynaptic basins integrating 2.22 e, and two disynaptic basins, V(C7,N8) and V′(C7,N8), integrating a total of 3.60 e. In addition, two disynaptic basins, V(N8, C(Me)) and V(N8, C′(Me)), integrating 1.88 e each, are observed.

On the other hand, ELF of Cp **1**, not represented in Figure 3, shows the presence of two disynaptic basins, V(C1,C2) and V′(C1,C2), integrating a total of 3.38 e, one V(C2,C3) disynaptic basin, integrating a total of 2.21 e, and two disynaptic basins, V(C3,C4) and V′(C3,C4), integrating a total of 3.38 e. The C1=C2–C3=C4 bonding region accumulated a total of 8.97 e.

The natural atomic charges are given in Figure 3. At iminium cation **6**, while the C5 carbon is positively charged by 0.15 e, the N6 nitrogen is negatively charged by 0.26 e. On the other hand, at conjugated iminium **18**, while the C5 and C6 carbons are negatively charged by 0.15 and 0.33 e, respectively, the C7 carbon is slightly positively charged by 0.25 e; the N8 nitrogen is negatively charged by 0.29 e.

Some interesting conclusions can be drawn from the analysis of the electronic structure of imines **13** and **14** and iminium cations **6** and **18**: (i) ELF of the C=C and C=N double bonds of imines **13** and **14** showed a depopulation of these bonding regions as a consequence of the polarization of the molecular electron density towards the N nitrogen; note that the non-bonding region of the nitrogen integrated more than 2.60 e; (ii) the populations of these bonding regions were higher at iminium cations **6** and **18** than at imines **13** and **14**. This finding is a consequence of the formation of the new N–C(Me) single bonds, which are populated by less charge of 1.92 e. Consequently, the excess of the non-bonding nitrogen electron density of imines **13** and **14** is redistributed towards the C–C and C–N bonding regions of **6** and **18**; (iii) the nitrogen centers of the iminium cations **6** and **18** are negatively charged, a behavior against the positive charge represented at the Lewis structures of iminium cations. This behavior is a consequence of the fact that in organic cationic species, the hydrogens are the centers which mainly support the positive charge; (iv) analysis of the electronic structures of the conjugated imine **14** and iminium cation **18** shows that the different substitution on the C5=C6 double bonds of these species does not substantially modify the electronic structure of the ethylene framework participating in these DA reactions; and finally, (v) at the most electrophilic iminium cation **18**, the β-conjugated C5 carbon, which supports a negative charge of −0.15 e, is the most electrophilic center of this cationic species; see analysis of the electrophilic Parr functions. This finding supports the earlier observation that the electrophilicity and nucleophilicity cannot be related only with charge distribution at the GS of the molecules, but with the propensity of changes on electron density resulting from the GEDT along a polar reaction, which is anticipated by the analysis of the Parr functions [28].

#### 2.1.2. Analysis of the CDFT Reactivity Indices at the GS of the Reagents

Many studied devoted to cycloaddition reactions have proven that the analysis of the global and local reactivity indices defined within the CDFT [18,19] is a powerful tool to understand polar reactions [36,37]. The CDFT indices were obtained at the B3LYP/6-31G(d) computational level because they were used to define the scales of electrophilicity and nucleophilicity [19]. The global reactivity indices of imines **13** and **14**, iminium cations **6**, **15**–**19**, and Cps **1** and **20** are collected in Table 1.

The electronic chemical potentials [18,38] *μ* of imines, *μ* = −3.46 (**13**) and −3.91(**14**) eV, are closer to that of Cp **1**, *μ* = −3.01 eV, indicating that the corresponding HDA reactions will have non-polar characteristics and high activation energies. On the other hand, the six iminium cations present very low electronic chemical potential *μ* values, between −9.53 (**18**) and −11.83 (**15**) eV. Consequently, the corresponding I-DA reactions will experience a high GEDT and very low activation energies.

The electrophilicity ω indexes [16] of imines **13** and **14** are 0.88 and 1.31 eV, respectively, being classified as marginal electrophiles, whereas the nucleophilicity *N* values [17], 2.26 and 2.30 eV, also classify them as moderate nucleophiles. The presence of the electronegative N8 nitrogen in the heterodiene **14** decreases its nucleophilic character with respect to that of 1,3-butadiene, *N* = 2.93 e. Consequently, imines **13** and **14** will not participate, either as ethylene or as diene in P-DA reactions.

The electrophilicity ω index of iminium cations, between 8.20 (**16**) and 9.53 (**17**) eV, classifies them as strong electrophiles. These high values, higher than 4.0 eV, classify them as superelectrophiles [21]. Consequently, they will participate in I-DA reactions with unappreciable activation energies. All iminium cations present negative nucleophilicity *N* indices.

The electrophilicity ω and nucleophilicity *N* indices of Cp **1**, 0.83 and 3.37 eV, respectively, classify it as a moderate electrophile and a strong nucleophile. The inclusion of an electron-releasing methyl group on the C1 carbon of Cp **1** decreases the electrophilicity ω index of CpMe **20** to 0.76 eV and increases its nucleophilicity *N* index to 3.62 eV, thus classifying it as a marginal electrophile and a strong nucleophile.

From the electrophilicity ω indices of the iminium cations given in Table 1, some interesting conclusions can be drawn: (i) the series of the conjugated iminium cations **17**–**19** are more electrophilic than iminium cations **6** and **16**. In spite of that, the latter have less electronic chemical potential *μ*; the softer character of the former makes them more electrophilic. Note that the softness, S, is the inverse of the hardness, η, S = 1/η; (ii) the protonated iminium cations are more electrophilic than the dialkyl derivative cations, due to the electron-releasing character of the CH_3_ and CH_2_ groups; and finally, (iii) the two cyclic iminium cations have different behaviors. The dimethyl derivative **6** is more electrophilic than cyclic **16**, whereas cyclic iminium cation **19** is more electrophilic than the dimethyl derivative **18**.

**Figure 4:**
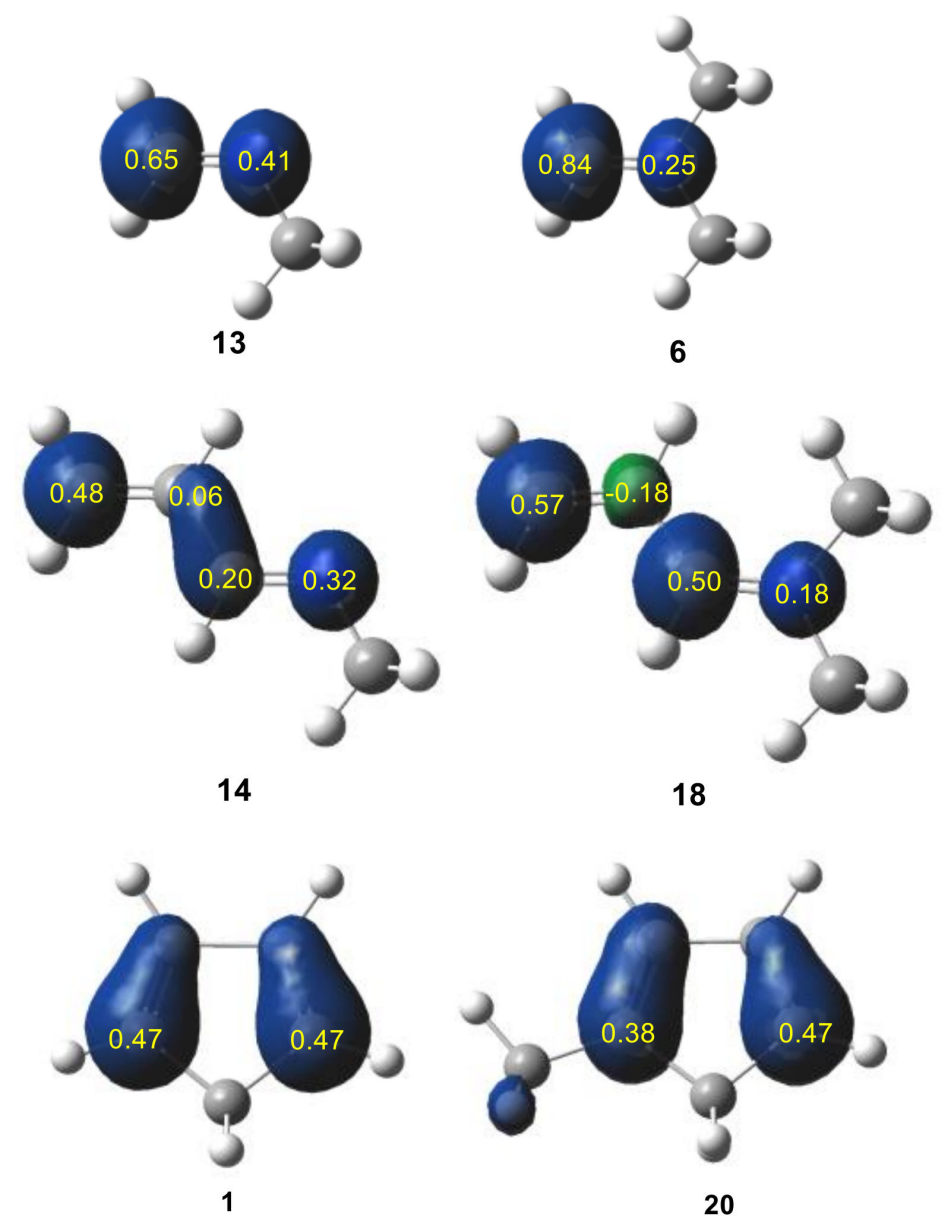
Three-dimensional representations of the Mulliken atomic spin densities of the radical anions of imines **13** and **14**, neutral radicals **6** and **18**, the radical cations of Cp **1** and CpMe **20**, and the electrophilic $P_{k}^{+}$ and nucleophilic $P_{k}^{-}$ Parr functions.

DA reactions involving non-symmetric electrophilic compounds take place via asynchronous TSs. The most favorable two-center interactions between the two interacting species in the polar process takes place between the most electrophilic center of the electrophile, and the most nucleophilic center of the nucleophile [39]. In this context, the electrophilic $P_{k}^{+}$ and nucleophilic $P_{k}^{-}$ Parr functions [28] derived from the excess of spin electron density reached via the GEDT [12] process from the nucleophile toward the electrophile have been shown to be one of the most accurate and insightful tools for the study of local reactivity in polar and ionic processes. The C1 and C4 carbons of Cp **7** are the most nucleophilic centers of this diene. In order to explain the asynchronicity in the C–C and C–N(C) bond formation, the electrophilic $P_{k}^{+}$ functions of imines **13** and **14**, and dimethyl iminium cations **6** and **18**, were analyzed. The corresponding values are given in Figure 4.

As expected, the C5 carbon of imine **13** and iminium cation **6**, $P_{k}^{+}$ = 0.65 and 0.84, respectively, are more electrophilically activated than the N6 nitrogen, $P_{k}^{+}$ = 0.41 and 0.25. Formation of the iminium cation **6** increases the electrophilic activation of the C5 carbon, indicating that the corresponding TS will be more asynchronous.

The C5 carbon of conjugated imine **14**, $P_{k}^{+}$= 0.48, is also the most electrophilic center of this species, following the N8 nitrogen, $P_{k}^{+}$ = 0.32. On the other hand, despite the strong electrophilic activation of the C7 carbon of iminium cation **18**, $P_{k}^{+}\;$= 0.50, the C5 carbon remains as the most electrophilic center of this species, $P_{k}^{+}\;$= 0.57. Note that the C6 carbon is electrophilically deactivated, at −0.18, indicating that the corresponding TS will be highly asynchronous.

Analysis of the electrophilic $P_{k}^{+}$ Parr functions of these species indicates that the most favorable two-center interactions at the corresponding TSs will take place between one of the two end carbons of the symmetric diene Cp **1**, i.e., the C1 carbon, and the C5 carbons of these imine derivatives. Formation of the corresponding iminium cation increases the electrophilic activation of the C5 carbon with respect to the N6 or C6 centers, increasing the asynchronicity in C–N(C) single bond formation.

It is interesting to remark that the electrophilic activation of the C5 carbon of iminium cation **18**, $P_{k}^{+}$ = 0.57, is higher than that of the C7 carbon, $P_{k}^{+}$ = 0.50, even though the C5 carbon is negatively charged, −0.15 e, whereas the C7 carbon is positively charged, 0.25 e (see Figure 3). This finding supports the earlier assumptions that electrophilicity is not controlled by positively charged centers [21,32].

Analysis of the nucleophilic $P_{k}^{-}$ Parr functions of Cp **1** indicates that the C1 and C4 are symmetrically activated. These carbons are the most nucleophilic center of Cp, $P_{k}^{-}\;$= 0.47. The inclusion of an ER-Me at the C1 carbon does not modify the nucleophilic $P_{k}^{-}$ Parr function at the C4 carbon, $P_{k}^{-}$ = 0.47, but relocates the nucleophilic $P_{k}^{-}$ Parr function at the C1 and C2 carbons. Consequently, the C4 carbon becomes the most electrophilic center of CpMe **20**.

### 2.2. Study of the Potential Energy Surface of I-DA Reactions

#### 2.2.1. Study of the I-DA Reactions of Iminium Cations **6**, **15**–**19** with Cp **1**

**Scheme 6:**
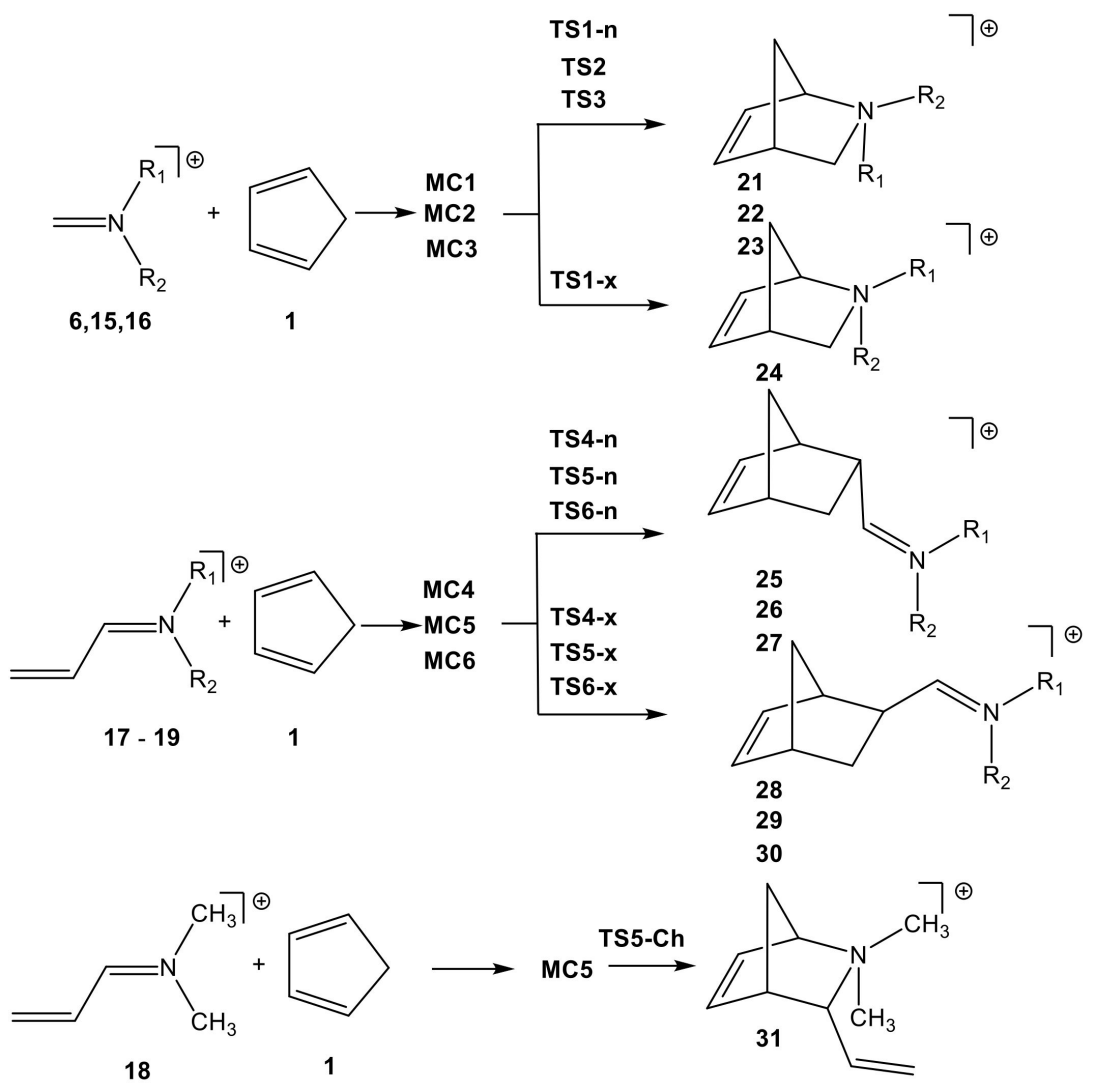
I-DA reactions of iminium cations **6**, **15**–**19** with Cp **1**.

For the symmetric iminium cations **6** and **16**, only one reaction path is feasible, whereas for the non-symmetric iminium cations **15**, **17**, **18** and **19**, two stereoisomeric reaction paths are feasible: the *endo* and the *exo*. In addition, due to the presence of two double bonds, C=C and C=N in the iminium cations derived from imine **14**, the chemoisomeric reaction path associated with the attack of Cp **1** to the C7=N8 double bond of iminium cation **18** was also considered (see Scheme 6). The stationary points involved in the six I-DA reactions are given in Scheme 6, and the relative energies in acetonitrile are given in Table 2. Note that due to the high instability of gas phase cationic species, the relative energies are analyzed in acetonitrile. Thus, whereas acetonitrile stabilizes the neutral Cp **1** by 7.7 kcal·mol^−1^, iminium cation **15** is stabilized by 53.7 kcal·mol^−1^. In order to understand the behaviors of these I-DA reactions, the DA reactions of imines **13** and **14** with Cp **7** were also studied (see Section 1 in the Supplementary Material).

At the beginning of the reactions, a series of molecular complexes could be found. They were characterized by the presence of weak electronic interactions between Cp **1** and the iminium cations. For each of these I-DA reactions, only the most stable MC was selected as the energy reference. The MCs were found to be between 5.6 (**MC4**) and 7.2 (**MC3**) kcal·mol^−1^ more stable than the separated reagents. The relative energies of the TSs with respect to the separated reagents associated with these I-DA reactions were found to be between −0.6 (**TS1-n**) and 4.4 (**TS4-x**) kcal·mol^−1^; the reactions were exothermic between 24.0 (**22**) and 28.4 (**21**) kcal·mol^−1^.

Some interesting conclusions can be drawn from the energy results given in Table 2: (i) the formation of MCs is an exothermic process, with energy less than 7.2 (**MC3**) kcal·mol^−1^. However, if the thermal correction and the entropies are considered, they become exergonic (see later); (ii) considering these MCs, the activation energies associated with these I-DA reactions were found to be between 6.0 (**TS1-n**) and 10.0 (**TS3**) kcal·mol^−1^. These activation barriers are between 13 and 20 kcal·mol^−1^ lower in energy that those associated with the DA reactions of neutral imines **13** and **14** with Cp **1** (see Scheme S1 in Supplementary Material); (iii) these I-DA reactions present low *endo* stereoselectivity; the energy differences between the *endo* and *exo* TSs are less than 1.2 kcal·mol^−1^ (**TS1-n**). The *endo* stereoselectivity decreases with the increase in the activation energy; (iv) the reaction of Cp **1** with iminium cation **18** is completely chemoselective, as **TS5-Ch** was observed to have an energy value 7.7 kcal·mol^−1^ above **TS5-n**. This high energy difference enables discarding the attachment of Cp **1** onto the C7=N8 double bond iminium cations **17**–**19**; (v) these I-DA reactions are highly exothermic, higher than 24.0 (**22**) kcal·mol^−1^; finally, (vi) the formation of **31** is exothermic by 15.3 kcal·mol^−1^. Consequently, the attachment of Cp **1** on the C7=N8 double bond iminium cation **18** is both kinetically and thermodynamically disfavored with respect to the attachment to the C5=C6 double bonds.

In P-DA reactions, the *endo* selectivity increases with the increase in the polar character of the reaction, resulting in the GEDT taking place at the TSs [13]. The GEDT increases the zwitterionic character of the TSs, increasing the favorable electrostatic interactions appearing between the ends of the two interacting frameworks along the *endo* approach mode. However, in I-DA reactions, the GEDT provokes a redistribution of the electron density; thus, positive charges appear at the two interacting frameworks, consequently presenting low *endo* stereoselectivity

**Figure 5:**
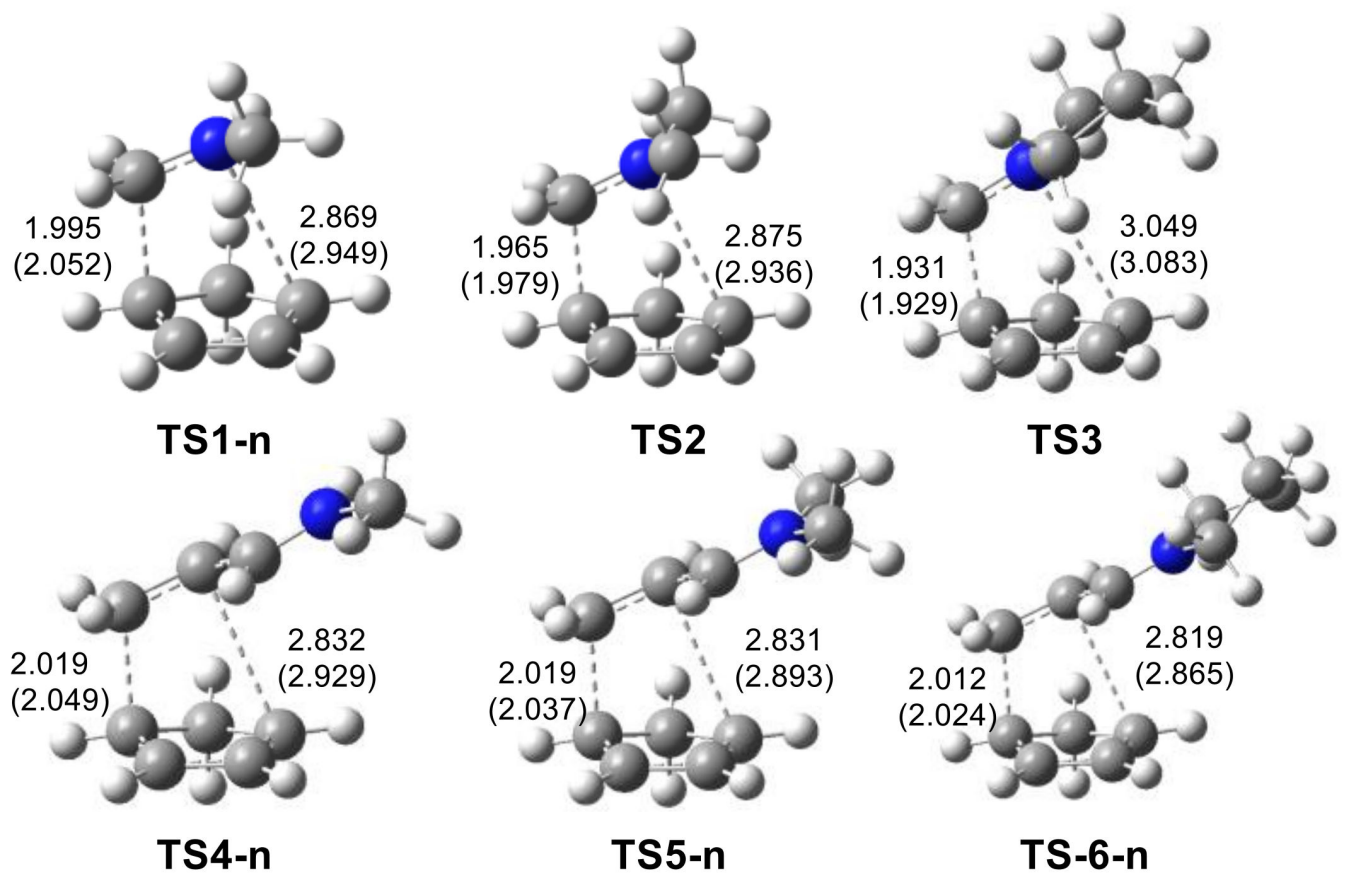
ωB97XD/6-311G(d,p) geometries of the *endo* TSs for DA reactions of iminium cations **6**, **15**–**19** with Cp **1**. The C–C and C–N distance are given in Angstroms. The gas phase distances are given in parentheses.

The geometries of the more favorable *endo* TSs associated with these I-DA reactions are given in Figure 5. The geometries of the *exo* TSs are given in Figure S4 in the Supplementary Material. The distances between the C1–C5 and C4–N6 interacting centers at the TSs of the I-DA reactions of iminium cations **6**, **15** and **16** in acetonitrile are: 1.995 and 2.869 Å at **TS1-n**, 1.965 and 2.875 Å at **TS2**, and 1.931 and 3.049 Å at **TS3**, whereas those between the C1–C5 and C4–C6 interacting centers at the TSs of the I-DA reactions of conjugated iminium cations **17**–**19** are: 2.019 and 2.832 at Å **TS4-n**, 2.019 and 2.831 Å at **TS5-n**, and 2.012 and 2.819 at Å **TS6-n**. Some interesting conclusions can be drawn from these geometrical parameters: (i) the distances indicate that these TSs are associated with highly asynchronous single bond formation processes. They are associated with a two-center interaction between the most nucleophilic center of Cp **1**, the C1 carbon, the most electrophilic center of these iminium cations, and the C5 carbon, a behavior anticipated by analysis of the electrophilic Parr functions; (ii) for the two series of I-DA reactions, there is a decrease in the C1–C5 distance with the increase in the activation energy; for these series, the most favorable TS is more delayed. This finding can be understood as an application of the Hammond principle [40]; finally, (iii) comparison of the gas phase and acetonitrile geometries indicates that there is not a remarkable change with the inclusion of solvent effects on the gas phase geometries. The differences of the C1–C4 distances at the six TSs are less than 0.06 Å. These results suggest that although the inclusion of solvent effects modifies substantially the energies, it does not change the electronic structure of the TSs (see later).

Herein, it is interesting to remark that unlike the P-DA reactions in which the TSs have a zwitterionic character resulting from the GEDT, at the TSs associated with I-DA reactions, both frameworks are positively charged. These behaviors have an important experimental consequence; in P-DA reactions, polar solvents accelerate the reactions by a better solvation of the zwitterionic TSs than reagents, whereas in I-DA reactions, solvent effects deaccelerate them by better solvation of the cationic reagents than TSs.

The ionic nature of these I-DA reactions was evaluated by computing the GEDT at the gas phase TSs. The computed GEDT values at the corresponding TSs, which flux from the nucleophilic Cp **1** to the superelectrophilic iminium cations, was: 0.40e (**TS1-n**), 0.43 e (**TS1-x**), 0.43 e (**TS2** and **TS3**), 0.37 e (**TS4-n**), 0.39 e (**TS4-x**), 0.38 (**TS5-n** and **TS5-x**), 0.38 e (**TS6-n**) and 0.37 (**TS6-x**). These very high values imply the high GEDT taking place at these I-DA reactions. Note that at the DA reactions involving neutral imines, the GEDT is lower than 0.15 e (see Section 1 in Supplementary Material). As expected, the GEDT values at the TSs associated with the I-DA reactions involving the most electrophilic iminium cations **6**, **15** and **16**, is slightly higher than those involving unsaturated iminium cations **17**–**18**. The GEDT values at the TSs in acetonitrile are very close to those obtained in the gas phase; thus, the GEDT values of **TS1-n** and **TS2** in acetonitrile are 0.41e and 0.43 e, respectively. As expected, in these I-DA reactions, the electron density fluxed from Cp **1** towards the iminium cations, these reactions being classified as the forward electron density flux (FEDF) [41].

**Figure 6:**
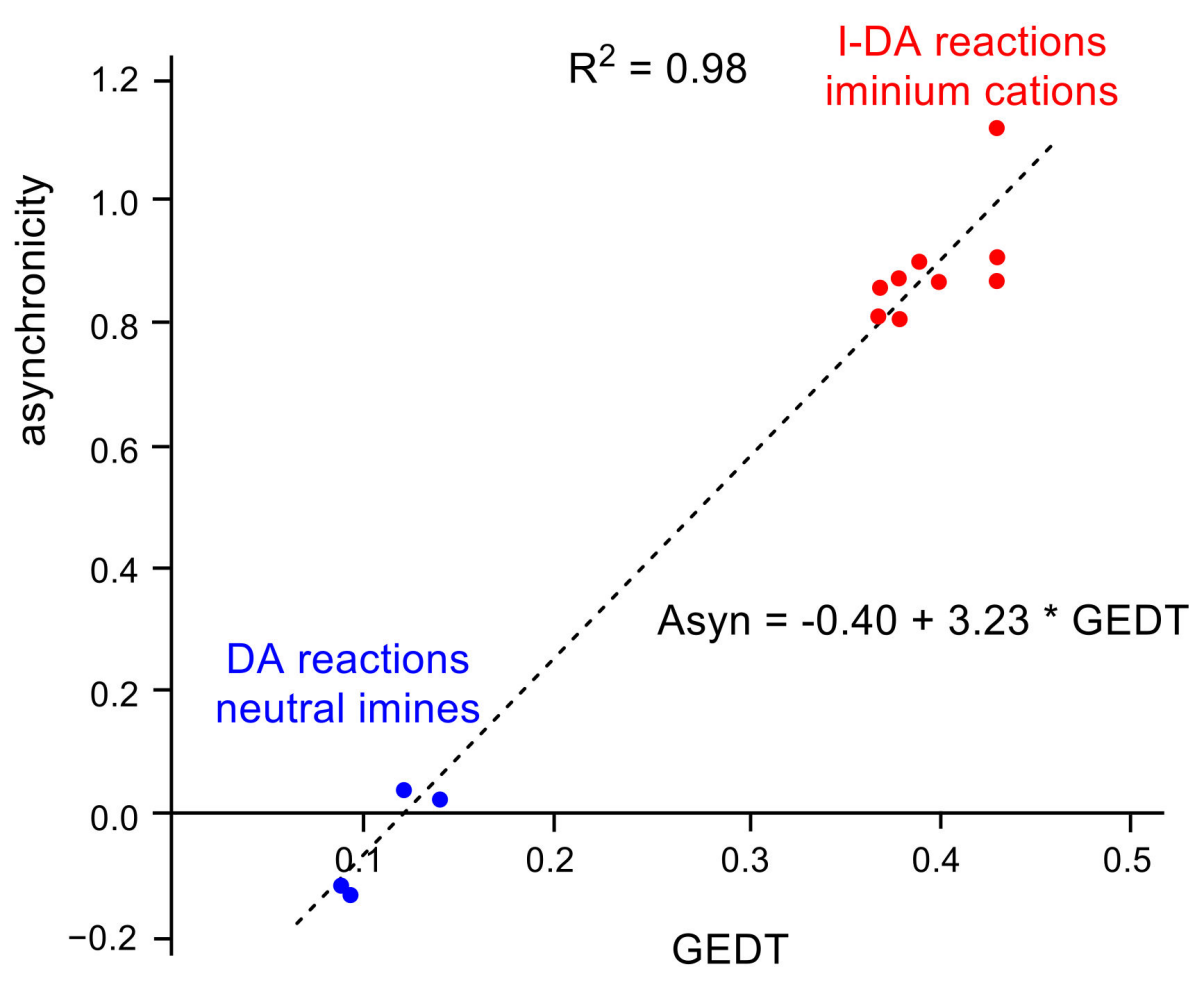
Plot of the asynchronicity, Asyn = (l1–l2) in Angstroms, vs. the GEDT, in e, taking place at the TSs associated with the DA reaction of non-symmetric imines and the I-DA reaction of non-symmetric iminium cations.

As shown Figure 6, a good linear correlation between the GEDT and the asynchronicity at the TSs of the DA reaction of imines **13** and **14** and the I-DA reaction of cations **6**, **15**–**19** can be established (*R*^2^ = 0.98). Thus, whereas the low polar DA reactions of neutral imines **13** and **14**, GEDT < 0.15 e, present low asynchronous TSs, Δl < 0.2 Å, the I-DA reactions of iminium cations **6**, **15**–**19** with GEDT > 0.37 e present highly asynchronous TSs, Δl > 0.8 Å. These behaviors are a consequence of fact that whereas low polar DA reactions of neutral imines are associated with low asynchronous four-center interactions, the I-DA reactions of non-symmetric iminium cations are associated with highly asynchronous two-center interactions. These findings account for the decrease in the steric (Pauli) repulsion with the increase in the ionic character of the reaction.

#### 2.2.2. Study of the Regioselectivity in I-DA Reactions

**Scheme 7:**
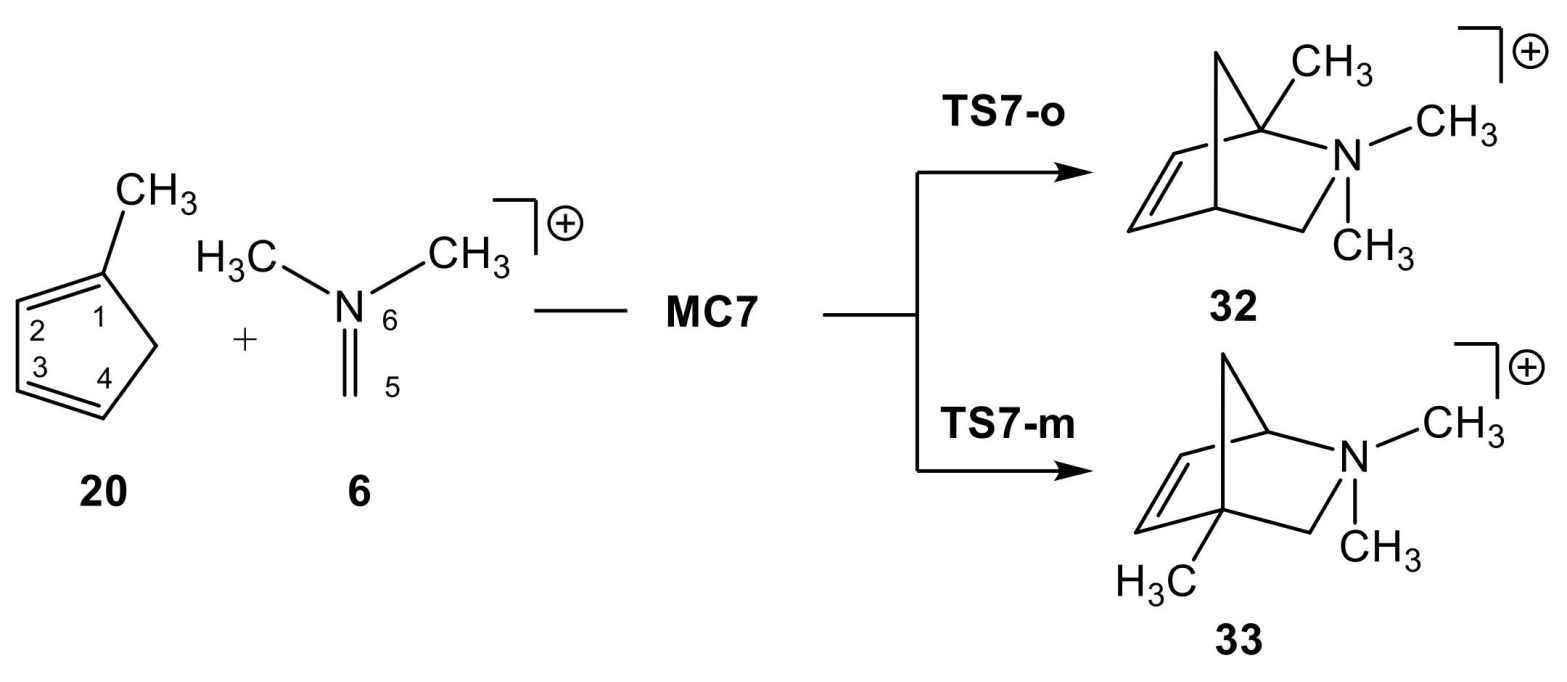
I-DA reaction of iminium cations **6** with CpMe **20**.

Due to the symmetry of Cp **1**, these I-DA reactions do not have regioisomeric reaction paths. In order to analyze the regioselectivity in I-DA reactions, the reaction of iminium cation **6** with CpMe **20** was also considered (see Scheme 7). Due to the symmetry of iminium cation **6**, only two competitive regioisomeric reaction paths, named *ortho* and *meta*, are feasible. The stationary points involved in this I-DA reaction are given in Scheme 7, whereas the relative energies in acetonitrile are given in Table 3.

The relative energies of the TSs with respect to the separated reagents are −0.8 (**TS7-o**) and 2.7 (**TS7-m**) kcal·mol^−1^; the reactions are exothermic by 24.3 (**32**) and 24.1 (**33**) kcal·mol^−1^. If the formation of **MC7** is considered, the activation energies associated with the two regioisomeric reaction paths become 6.7 (**TS7-o**) and 10.2 (**TS7-m**) kcal·mol^−1^. Consequently, this I-DA reaction is completely regioselective, because **TS7-m** was found to be 3.5 kcal·mol^−1^ above **TS7-o**. The activation energy associated with this I-DA reaction is 2.8 kcal·mol^−1^ lower in energy than that involving Cp **1** via **TS2**, as a consequence of the more nucleophilic character of CpMe **20** than Cp **1** (see Table 1 and Table 3).

**Figure 7:**
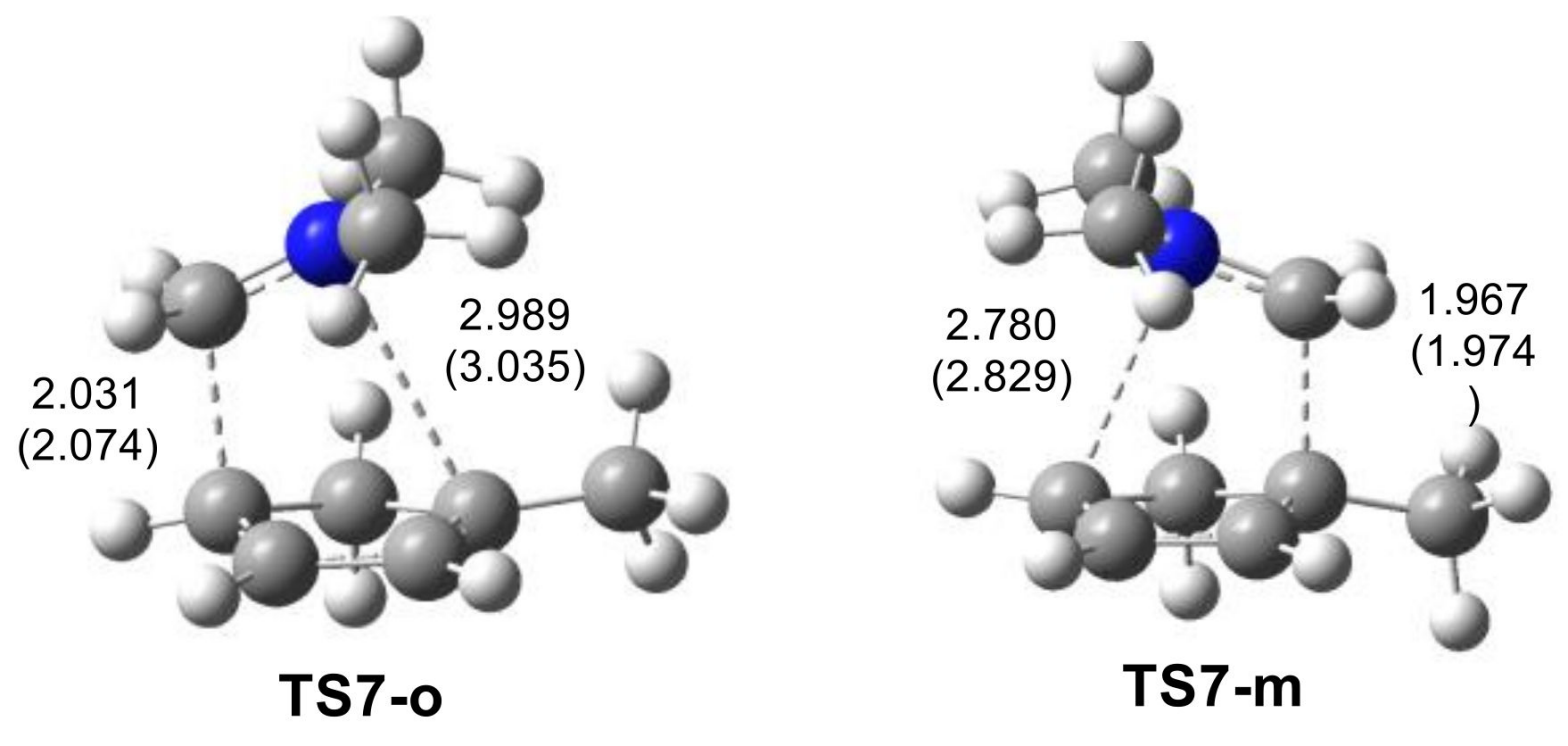
ωB97XD/6-311G(d,p) geometries of two regioisomeric TSs for I-DA reaction of iminium cation **6** with CpMe **20**. The distances are given in Angstroms. The gas phase distances are given in parentheses.

The geometries of the two regioisomeric TSs associated with this I-DA reaction are given in Figure 7. The distances between the C1–C5 and C4–N6 interacting centers at the TSs of the I-DA reaction of iminium cation **6** with CpMe **20** in acetonitrile are 2.031 and 2.989 Å at **TS7-o**, and 1.967 and 2.780 Å at **TS7-m**. These distances indicate that these TSs are associated with highly asynchronous single bond formation processes which are controlled by the most electrophilic C5 carbon of iminium cation **6**. The more favorable regioisomeric **TS7-o** is associated with the two-center interaction between the most electrophilic center of iminium cation **6** and the most nucleophilic center of CpMe **20**, the non-substituted C4 carbon, a behavior anticipated by the analysis of the Parr functions (see Figure 4).

At the two regioisomeric TSs, the GEDT taking place from the Cp framework to the iminium cation is 0.40 e at **TS7-o** and 0.44 e at **TS7-m**. These very high values point out the highly ionic character of this I-DA reaction. The GEDT at the more unfavorable **TS7-m** is higher than that at **TS7-o** as a consequence of the more advanced character of the former [42]. On the other hand, the high GEDT taking place at the TSs involving the non-symmetric iminium cation **6** accounts for the high asynchronicity found in this I-DA reaction.

#### 2.2.3. Thermodynamic Analysis of the I-DA Reaction of Iminium Cation **6** with Cp **1**

**Figure 8:**
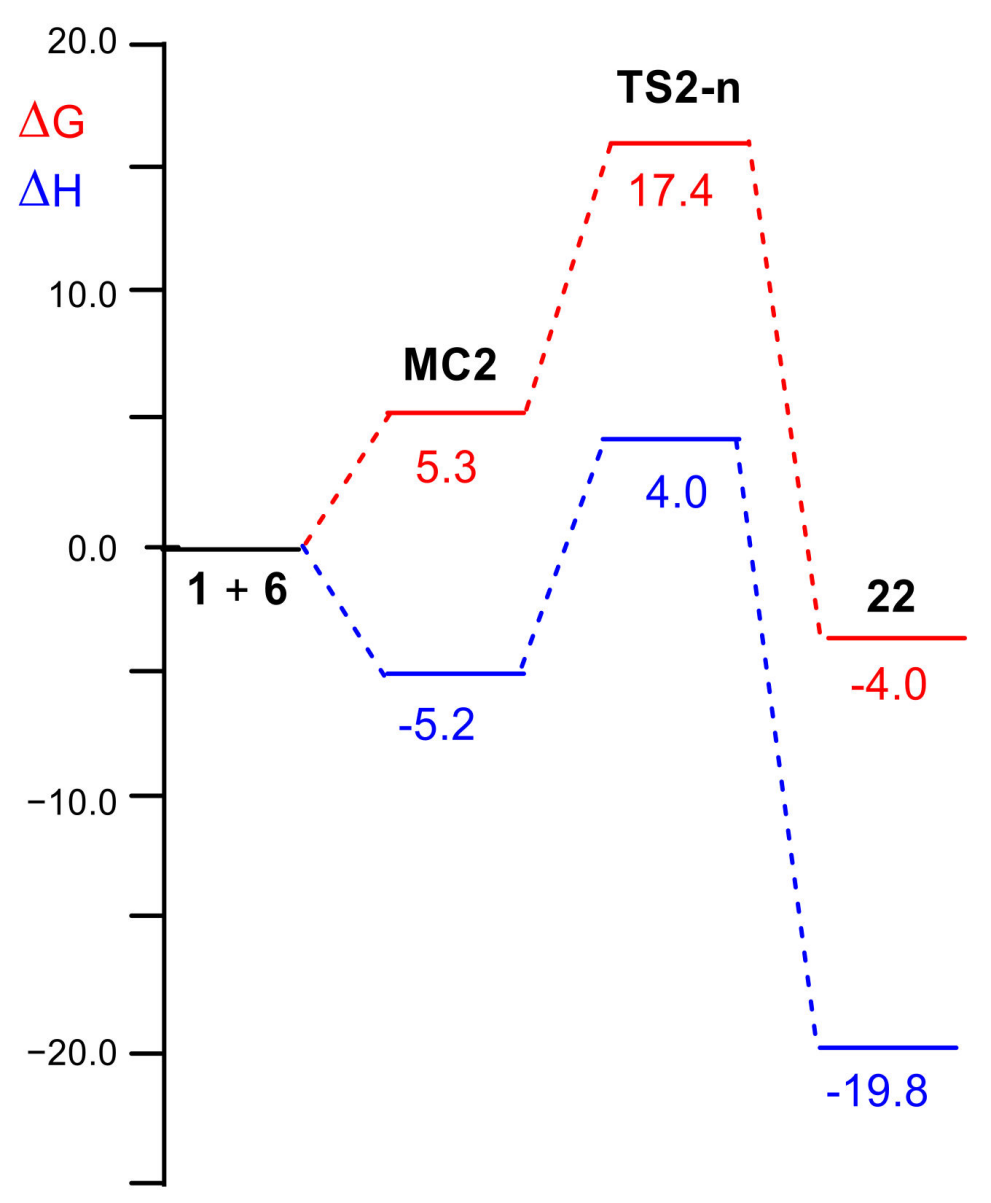
ωB97XD/6-311G(d,p) enthalpy, in blue, ΔH in kcal mol^–1^ and Gibbs free energy, in red, ΔG in kcal mol^–1^ profiles in acetonitrile at 25 °C, for the I-DA reaction of iminium cation **6** with Cp **1**.

The thermodynamic data of the reaction of iminium cation **6** with Cp **1**, as a representative I-DA reaction of this series, were analyzed. Relative enthalpies and Gibbs free energies, computed at 25 °C in acetonitrile, are given in Figure 8.

Inclusion of the thermal corrections with the electronic energies in acetonitrile decreases the relative enthalpies by between 1.1 and 4.2 kcal·mol^−1^. The lower incidences take place in the relative enthalpy of **TS2** which decreases by 1.1 kcal·mol^−1^ with respect to the electronic energy in acetonitrile. The inclusion of the thermal correction and entropies in enthalpies increases the relative Gibbs free energies by between 10.5 and 15.9 kcal·mol^−1^ as a consequence of the unfavorable activation entropy associated with these bimolecular processes, between −35.2 and −53.3 cal·mol^−1^·K^−1^. Thus, the activation Gibbs free energy associated with the I-DA reaction of iminium cations **6** with Cp **1** in acetonitrile rises to 17.4 kcal·mol^−1^, while the formation of **22** becomes exergonic by only −4.0 kcal·mol^−1^. It is important to remark that although the formation of **MC2** is an exothermic process, it is endergonic as a consequence of the unfavorable positive entropy associated with its formation (see Figure 8).

### 2.3. Topological Analysis of the Bonding Changes along the I-DA Reactions

MEDT establishes that the energy costs for bonding changes along a chemical reaction are responsible for its feasibility [9]. On the other hand, the comparative topological analysis of the electronic structures of TSs involved in the related reactions enables characterization of the similitudes or differences between them.

#### 2.3.1. ELF Analysis of the C–C and N–C Single Bond Formation along the I-DA Reaction of Cp **1** with Iminium Cation **6**

In order to understand the bonding changes along I-DA reactions, a BET study of the I-DA reaction of Cp **1** with iminium cation **6** was performed. The detailed BET study of this I-DA reaction is given in Section 2 in the Supplementary Material. Some appealing conclusions can be obtained from this BET analysis: (i) the energy cost (EC) associated with formation of **TS2**, which is found at *Phase III*, 2.2 kcal·mol^−1^, is mainly associated with the depopulation of the C5–N6 bonding region of the iminium moiety from 3.65 to 3.10 e. Thus, the activation energy of this I-DA reaction can be related to the EC associated with the rupture of the C5=N6 double bond of iminium cation **6**; (ii) the bonding changes associated with this I-DA reaction are very similar to those found in P-DA reactions, but they are more advanced in I-DA reactions; note that in P-DA reactions the formation of the first C–C single bond usually takes place afterwards to pass the TSs, whereas in this I-DA reaction, it takes place before to reach **TS2**. Note that at **TS2**, the C1–C5 single bond is already formed; (iii) although the C5 carbon of iminium cation **6** is the most electrophilic center of this molecule, the electron density coming from both the initial depopulation of C5–N6 bonding region and that of the GEDT is mainly accumulated at the N6 nitrogen first. Likewise, the bonding changes at the Cp framework are non-symmetric, and the changes in the C1–C2 bonding region are more advanced than those in the C3-C4 region. This behavior is a consequence of changes in electron density demanded for the formation of the first C1–C5 single bond; (iv) formation of the first C1–C5 single bond takes place at **TS2**, at a C–C distance of ca. 1.98 Å and with an initial population of 0.76 e, by sharing the non-bonding electron density of two C1 and C5 *pseudoradical* centers (62:38) created from the rupture of the corresponding multiple bonds along the reaction path before reaching the TS [12]; (v) formation of the second C4–N6 single bond takes place at a C–N distance of ca. 2.11 Å and with an initial population of 1.71 e, mostly through the donation of the non-bonding electron density of the N6 nitrogen developed from the rupture of the C5=N6 double bond of **6** to the C4 carbon (96:4); finally, (vi) this highly asynchronous I-DA reaction takes place through a marked non-concerted *two-stage one-step* mechanism [25], in which the formation of the second C4–N6 single bond takes place when the formation of the first C1–C5 bond is almost completed (94%).

#### 2.3.2. Comparative ELF Analysis of the TSs Involved in the P-DA Reaction of Cp **1** with Imines **13** and **14**, and Those Involved in the I-DA Reaction of Cp **1** with Iminium Cations **6** and **18**

**Figure 9:**
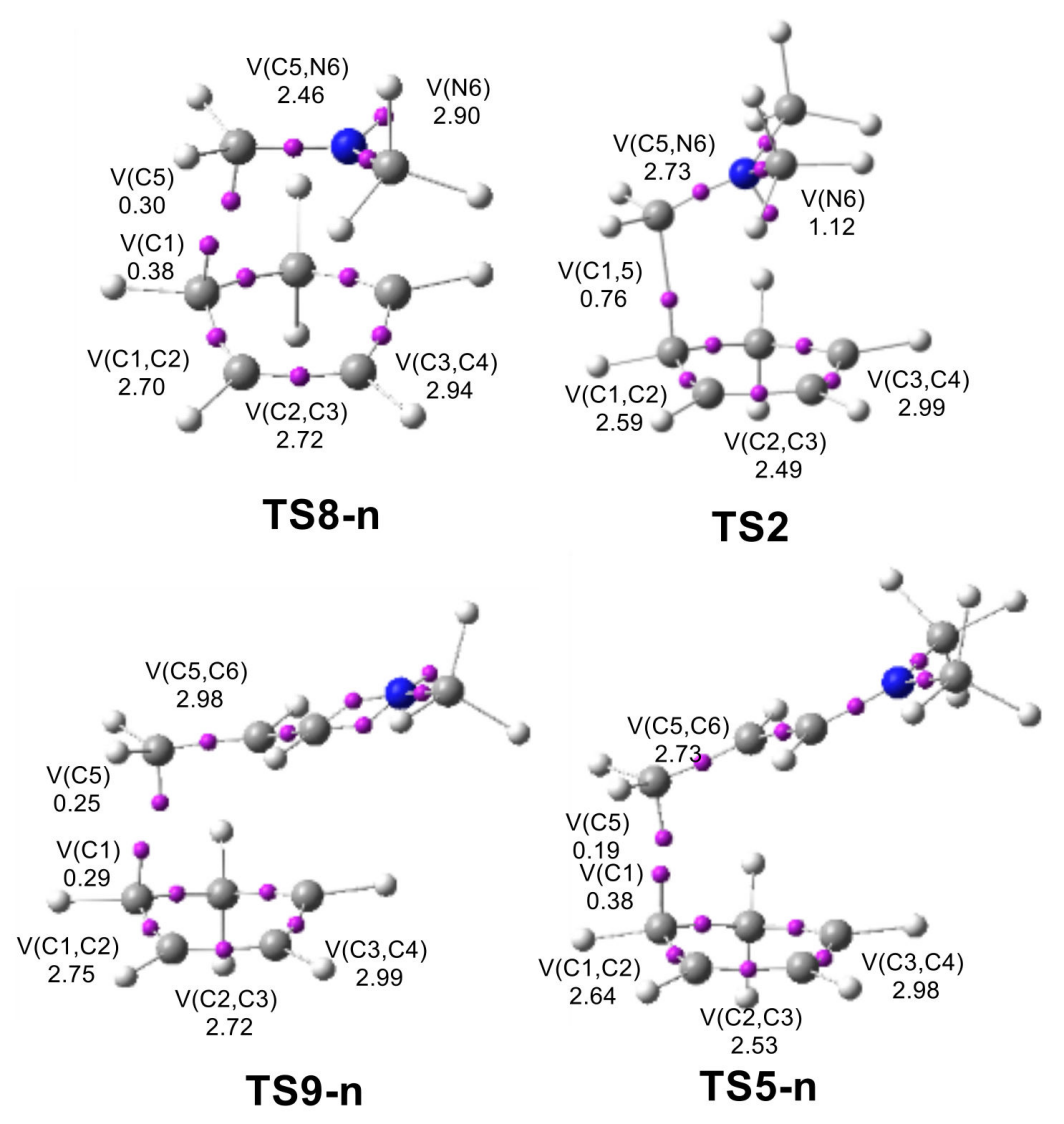
ELF basin attractor positions with the most significant valence basin populations of the TSs involved in the P-DA reaction of Cp **1** with imine **13**, **TS8-n**, unsaturated imine **14**, **TS9-n**, and those involved in the I-DA reaction of Cp **1** with iminium cation **6**, **TS2**, and unsaturated iminium cation **18**, **TS5-n**.

Finally, a comparative ELF topological analyses of the TSs involved in the P-DA reaction of Cp **1** with imine **13**, **TS8-n**, unsaturated imine **14**, **TS9-n**, and those involved in the I-DA reaction of Cp **1** with iminium cation **6**, **TS2**, and unsaturated iminium cation **18**, **TS5-n**, were performed in order understand the changes at the TSs of I-DA reactions with respect to those participating in P-DA reactions. The ELF attractors, with the most significant valence basin populations, are given in Figure 9.

The comparative analysis of the ELF of the polar **TS8-n**, **TS9-n** and ionic **TS5-n** indicates that they present great similarity. They show the presence of one V(C1) monosynaptic basin, integrating 0.38 e, 0.29 e and 0.38 e, respectively, and one V(C5) monosynaptic basin, integrating 0.30 e, 0.25 e and 0.19 e. These monosynaptic basins, which have been created along the reaction paths, are required for the subsequent C1–C5 single bond formation [12]. At these TSs, one V(C5,C[N]6) disynaptic basin is observed, with populations of 2.46 e, 2.98 e and 2.73 e, respectively, as a consequence of a slight depopulation of the C–C[N] bonding region. On the other hand, the C1–C2–C3–C4 bonding regions of these TSs are characterized by the presence of three disynaptic basins, V(C1,C2), V(C2,C3) and V(C3,C4), integrating totals of 8.36 e (**TS8-n**), 8.46 (**TS9-n**) and 8.15 e (**TS5-n**), respectively. A significant part of the electron density lost in these frameworks, with respect to that of Cp **1**, 8.97 e, is involved in the formation of the V(C1) monosynaptic basins present at these TSs.

Different behavior is observed at **TS2**. Instead of the two V(C1) and V(C5) monosynaptic basins present at the other three TSs, a new V(C1,C5) disynaptic basin, integrating 0.76 e, is observed. The presence of this disynaptic basin indicates that at **TS2**, the formation of the new C1–C5 single bond has begun already. Considering that the formation of the C–C single bonds takes place in the range of 2.0–1.9 Å [12], this different behavior of **TS2** with respect to the other three TSs is a consequence of its more advanced character. On the order hand, the C1–C2–C3–C4 and C5–C6 bonding regions of **TS2** are very similar to those at **TS5-n**.

From this ELF comparative analysis, it is possible to conclude that the four TSs belonging to P-DA reactions of imines and I-DA reactions of iminium cations present similar electronic structures. Considering the similarity of the ethylenic C5-C[N]6 regions of these TSs, the very low activation energies associated with these I-DA reactions with respect to those of the P-DA reactions of imines **13** and **14**, between 15 and 23 kcal·mol^−1^, can be related to the high GEDT taking place at the TSs of these I-DA reactions which favor the changes of electron density along the corresponding reaction paths [43].

#### 2.3.3. Analysis of the Origin of the Regioselectivity in I-DA Reactions

**Figure 10:**
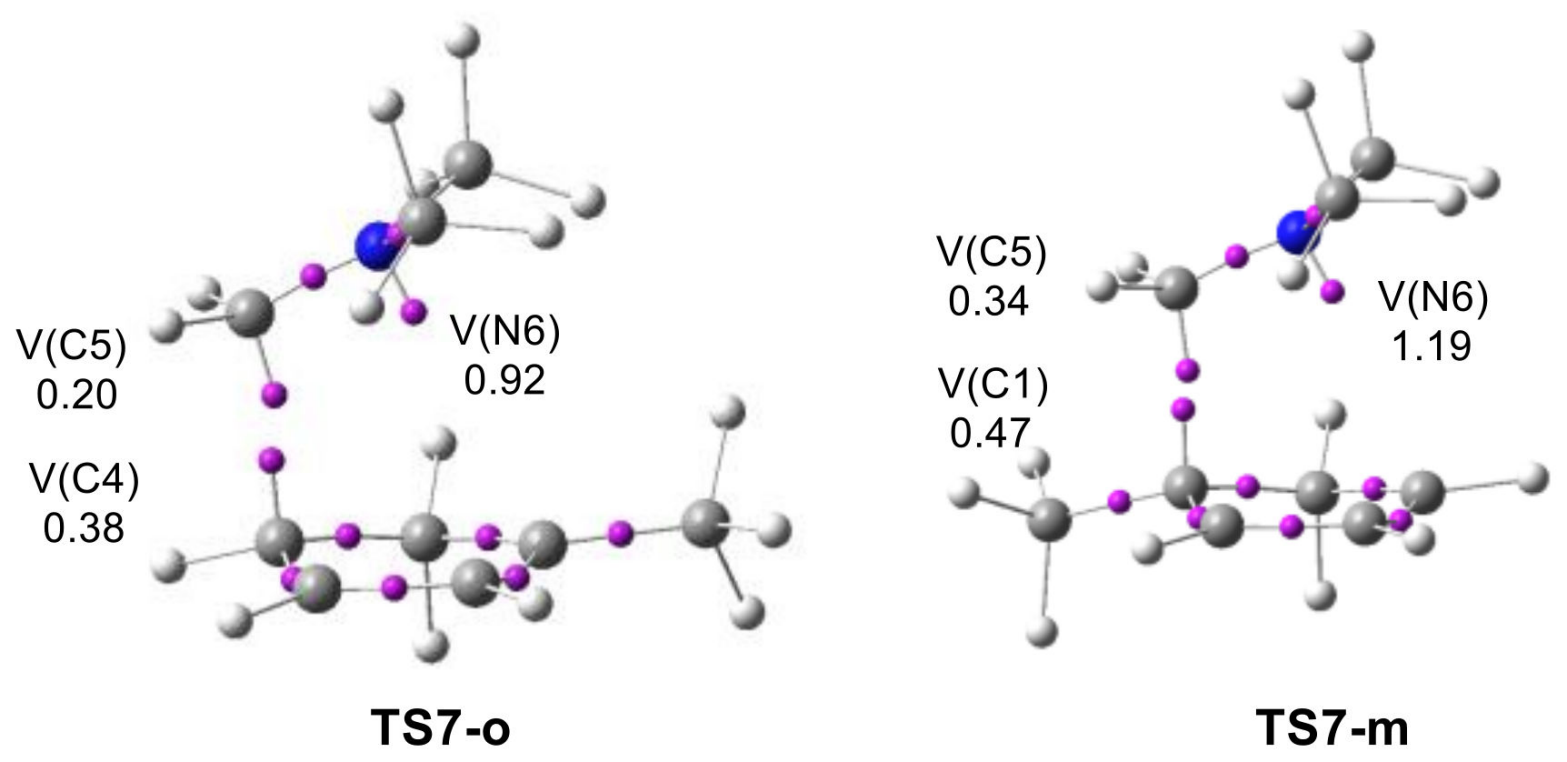
ELF basin attractor positions with the most significant valence basin populations of the regioisomeric TSs **TS7-o** and **TS7-m** involved in the I-DA reaction of CpMe **20** with iminium cation **6**.

Many theoretical studies of cycloaddition reactions participating non-symmetric electrophilic species have established that the most favorable reaction path is that involving the two-center interaction between the most nucleophilic center of the nucleophile and the most electrophilic center of the electrophile species [39]. These favorable interactions, which facilitate the creation of the first C–C single bonds, control the regio- and the chemoselectivity in polar reactions as well as the asynchronicity in single bond formation. In order to assert these finding in I-DA reactions, a topological analysis of the regioisomeric **TS7-o** and **TS7-m** was performed. The ELF attractors, with the most significant valence basin populations, are given in Figure 10.

As can be seen, both regioisomeric TSs present a similar ELF structure. At the iminium cation framework, both TSs present two monosynaptic basins, V(C5) and V(N6), integrating 0.20 e and 0.92 at **TS7-o** and 0.34 and 1.19 e at **TS7-m**, respectively. Note that the populations of these monosynaptic basins are higher at the more unfavorable **TS7-m** because it is more advanced (see Figure 10). On the other hand, the populations of the V(C5,N6) disynaptic basins at **TS7-o**, 2.91 e, and at **TS7-m**, 2.65 e, indicate that the C5–N6 bonding region of iminium cation **6** has experienced strong depopulations in these regions to create the V(N6) monosynaptic basin. Note that the population of this bonding region at iminium cation **6** is 3.48 e.

At the diene frameworks, whereas the most favorable **TS7-o** shows the presence of a V(C4) monosynaptic basin, integrating 0.38 e, at the most nucleophilic C4 carbon of CpMe **20** (see Figure 10), **TS7-m** presents a V(C1) monosynaptic basin, integrating 0.47 e at the substituted C1 carbon. On the order hand, the total electron density of the C1–C2–C3–C4 bonding region of the Cp framework is 8.2 e at **TS7-o** and 8.1 e at **TS7-m**. Note that the total electron density of this bonding region at CpMe **20** is approximately 9.0 e.

Consequently, at the more favorable **TS7-o**, the V(C4) monosynaptic basin required for the asynchronous formation of the first C4–C5 single bond is created at the most electrophilic center of CpMe **20**, the C4 carbon, which corresponds to the carbon of CpMe **20** that loses the least electron density along the GEDT process, a behavior anticipated by analysis of the nucleophilic $P_{k}^{-}$ Parr functions (see Figure 4) [28].

### 2.4. Origin of the Asynchronicity in I-DA Reactions

Several studies have explored the origin of the high asynchronicity found in the single bond formation in polar cycloaddition reactions [14,39]. These studies have emphasized that the high asynchronicity is a consequence of the two-center interactions between the most nucleophilic center of the nucleophile and the most electrophilic center of the non-symmetrically substituted electrophilic species [14,39]. Note that many highly asynchronous TSs associated with P-DA reactions have been associated with Michael-type additions [42,44].

**Figure 11:**
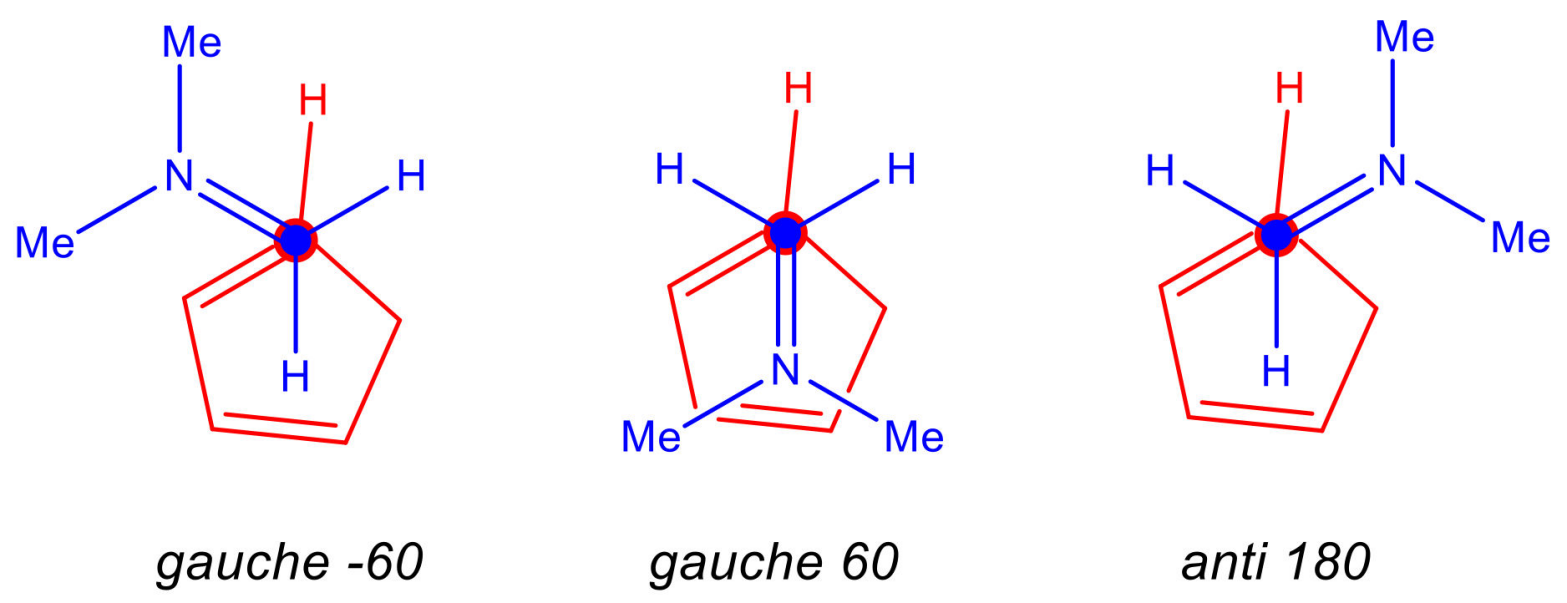
Three feasible stereoisomeric reaction paths associated with the C4 and C5 two-center interaction in the I-DA reaction of Cp **1** with iminium cation **6**.

Highly P-DA and I-DA reactions are associated with two-center interactions; therefore, the two terminal centers that do not participate in the formation of the first single bond do not interact. Note that at highly asynchronous TSs, the C4 carbon of Cp **1** remains sp^2^-hybridized. As a consequence, along the approach of the superelectrophilic iminium cations to the nucleophilic Cp **1**, three stereoisomeric TSs can be feasible in I-DA reactions: two *gauche* and one *anti* (see Figure 11). They are related to the C2–C1–C5–C(N)6 dihedral angle at the TSs; thus, **TS2**, which is associated with the *gauche 60* approach mode, presents a C2–C1–C5–N6 dihedral angle of 68.4 degrees.

**Figure 12:**
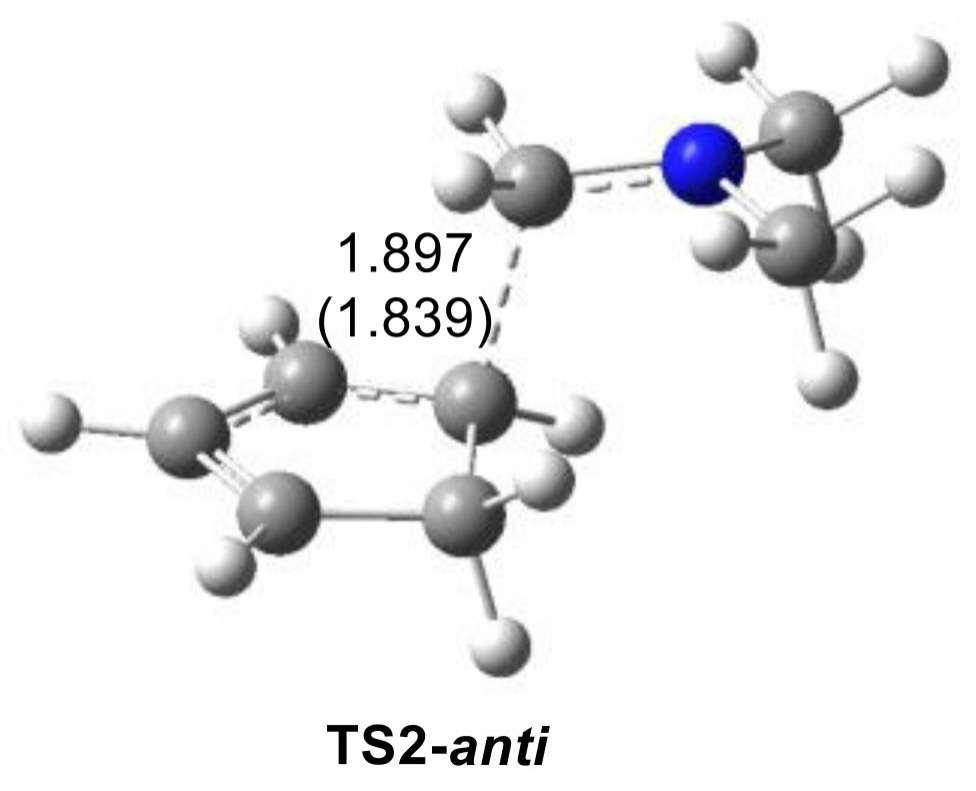
ωB97XD/6-311G(d,p) geometry of the stereoisomeric **TS2-*anti*** for the I-DA reaction of iminium cation **6** with Cp **1**. The distances are given in Angstroms. The gas phase distance is given in parenthesis.

Thus, along the approach of Cp **1** to iminium cation **6**, it is possible to find the *anti 180* TS, **TS2-*anti***, in which the C2–C1–C5–N6 dihedral angle is −162.3 degrees (see Figure 12). The corresponding stepwise *anti 180* reaction path is not competitive with the one-step *gauche 60* path, because **TS2-*anti*** is found 3.6 kcal·mol^−1^ above **TS2**. The GEDT at **TS2-*anti***, 0.48 e, is higher than that at **TS2**, 0.43 e, as a consequence of the shorter distance between the two frameworks at **TS2-*anti***, 1.839 Å. Note that the GEDT depends on the electrophilic/nucleophilic character of the reagents and the distance between the two interacting frameworks [42].

**TS2** and **TS2-*anti*** are two stereoisomeric species. However, whereas **TS2** is associated with a highly asynchronous TS, the stereoisomeric **TS2-*anti*** cannot be characterized as a highly asynchronous TS because it is associated with the first step of a stepwise I-DA reaction. These stereoisomeric TSs are associated with a similar C1–C5 single bond formation process, yielding cycloadduct **22** (see Scheme 6). The unique relevant differences between both stereoisomeric TSs are the arrangement of the non-bonding electron density of the N6 nitrogen (see Figure S5 in Supplementary Material). Interestingly, whereas at the *gauche 60* **TS2** the two methyl groups of the iminium cation **6** are located above the hydrocarbon ring of Cp **1**, at the *anti 180* **TS2-*anti***, they are farther away.

It appears that the favorable electronic interactions present between the non-bonding electron density created at the N6 nitrogen along the reaction path and the C2–C3–C4 allylic framework of Cp **1**, which is positively charged, and not the steric repulsions associated with steric (Pauli) repulsions between the two interacting frameworks, which will be higher at the more favorable *gauche 60* **TS2** than at *anti 180* **TS2-*anti***, such has been suggested [29], will be responsible for the higher stability of **TS2** than **TS2-*anti***. Consequently, it appears that steric repulsions do not control anything.

It can be concluded that the observed decrease in the steric repulsion with the increase in the nucleophilic/electrophilic interactions is a consequence of the increase in the asynchronicity resulting from an increase in the favorable two-center interactions taking place at the polar and ionic TSs involving asymmetric electrophilic species caused by the GEDT. Therefore, the reduction in the steric (Pauli) repulsions characterized by Bickelhaup et al. after performing EDA [29], is a consequence of the increase in the asynchronicity, which increases the distance between the ends of the two interacting frameworks, and never vice versa.

## 3. Conclusions

The behaviors of the I-DA reactions have been investigated within the MEDT at the ωB97XD/6-311G(d,p) computational level. To this end, the P-DA reactions of two imines **13** and **14**, and the I-DA reactions of six iminium cations **6**, **13**–**19** with Cp **1** have been investigated.

ELF topological analysis of these aza derivatives shows that the double bonds of both imines and iminium cations are very similar. Interestingly, the populations at the double bonds of iminium cations are slightly higher than at imines as a consequence of the reorganization of the non-bonding electron density of the N nitrogen present in imines.

The superelectrophilic character of iminium cations, ω > 8.20 eV, accounts for the high reactivity of these species with respect to that of the corresponding neutral imines, ω < 1.31 eV, participating in P-DA reactions. Analysis of the electrophilic $P_{k}^{+}$ Parr functions at the iminium cations characterizes the C5 carbons as the most electrophilic center of these species, thus accounting for the high regioselectivity and high asynchronicity found in these I-DA reactions.

The activation energies of the I-DA reactions were found to be between 13 and 20 kcal·mol^−1^ lower in energy than those associated with the P-DA reactions of neutral imines. These I-DA reactions are low *endo* selective as a consequence of the cationic character of the TSs, but highly regioselective.

Unlike P-DA reactions, polar solvents have a poor effect on the relative energies, and an unappreciable effect on the geometries. Interestingly, in solution, the activation energies increase slightly as a consequence of the better solvation of the iminium cations than TSs.

ELF topological analysis of the bonding changes along the I-DA reaction of iminium cation **6** with Cp **1** shows that C–C single bond formation takes place at **TS2** at a C–C distance of ca. 1.98 Å, and with an initial population of 0.76 e, by sharing the non-bonding electron density of two C1 and C5 *pseudoradical* centers (62:38) created from the rupture of the corresponding multiple bonds along the reaction path before reaching the TS. These centers correspond with the most electrophilic C5 carbon of iminium cation **6**, and to the most nucleophilic C1 carbon of Cp **1**, behaviors anticipated by analysis of the Parr functions. Interestingly, the pattern found for the C–C bond formation in this I-DA reaction is very similar to that found in N-DA and P-DA reactions [12].

Finally, an ELF comparative analysis of the TSs involved in the P-DA reactions of imines and those participating in the I-DA reactions of iminium cations showed that there are a great similarity in the bonding changes in both polar and ionic TSs. The main difference was found in the GEDT taking place in ionic reactions, higher than 0.37 e; the high GEDT taking place in I-DA reactions markedly decreased the activation energies to diminish the ECs associated with the changes in electron density required to determine the electronic structure of the TSs.

Well established concepts in organic chemistry, such as electrophilicity and nucleophilicity, are used within MEDT to explain and even to predict the chemical reactivity of iminium cations participating in I-DA reactions. On the other hand, the high GEDT taking place in the cationic TSs explains the low *endo* selectivity and low solvent effects found in I-DA reactions. The present MEDT study establishes that the high GEDT taking place at the TSs of the I-DA reactions resulting from the superelectrophilic character of the iminium cations is responsible for the features of these types of DA reactions. This contrasts with the steric (Pauli) repulsions origin proposed recently by Bickelhaup et al. [29,45] based on an ASM analysis, which is markedly different from the MEDT.

## 4. Computational Details

DFT calculations were performed using the hybrid ωB97X-D function [46], which includes long range exchange (denoted by X) correction as well as the semiclassical London-dispersion correction (indicated by suffix-D). The standard 6-311G(d,p) basis set was used [47], which includes d-type polarization for second-row elements and p-type polarization functions for hydrogen atoms. The Berny method was used in optimizations [48,49]. Only one imaginary frequency characterized all studied TSs. The intrinsic reaction coordinate (IRC) paths [50] were explored to find the unique connections given between the TSs and the minimum stationary points [51,52]. Solvent effects of acetonitrile were considered by full optimization of the gas phase structures at the same computational level using the polarizable continuum model [53,54] (PCM) in the framework of the self-consistent reaction field [55,56,57] (SCRF).

The GEDT [12] values were estimated by an NPA [34,35], using the equation GEDT (f) = $\Sigma_{q\in f}q$, where q is the charge at atoms of a framework (*f*) at the TSs. CDFT reactivity indices [18,19] were calculated through the equations given in [19].

All calculations were carried out with the Gaussian 16 suite of programs [58]. The topology of the ELF [33] of the ωB97XD/6-311G(d,p) monodeterminantal wave functions was assessed using the TopMod [59] package with a cubical grid of step size of 0.1 Bohr. The GaussView program [60] was used to visualize molecular geometries of all the systems as well as the position of the ELF basin attractors. The ELF localization domains at an isovalue of 0.75 a.u. were obtained with Paraview software [61,62].

## Figures and Tables

**Table 1:** B3LYP/6-31G(d) Global reactivity indices, the electronic chemical potential, μ, chemical hardness, η, electrophilicity, ω, and nucleophilicity, *N*, in eV, of imines **8** and **9**, iminium cations **1**–**6**, and Cp **7**.

	μ	η	ω	*N*
**17**	−10.11	5.36	9.53	−3.67
**15**	−11.83	7.80	8.97	−6.61
**19**	−9.76	5.32	8.95	−3.30
**18**	−9.53	5.22	8.71	−3.02
**6**	−11.18	7.56	8.26	−5.84
**16**	−10.51	6.73	8.20	−4.75
**14**	−3.91	5.84	1.31	2.30
**13**	−3.46	6.80	0.88	2.26
Cp **1**	−3.01	5.48	0.83	3.37
CpMe **20**	−2.84	5.31	0.76	3.62

**Table 2:** ωB97XD/6-311G(d,p) relative electronic energies in acetonitrile, kcal·mol^−1^, of the stationary points involved in the I-DA reactions of iminium cations **6**, **15**–**19** with Cp **1**.

	**15**		**6**		**16**
**MC1**	−6.6	**MC2**	−6.6	**MC3**	−7.2
**TS1-n**	−0.6	**TS2**	2.9	**TS3**	2.8
**TS1-x**	0.6				
**21**	−28.4	**22**	−24.0	**23**	−24.6
**24**	−28.1				
	**17**		**18**		**19**
**MC4**	−5.6	**MC5**	−5.6	**MC6**	−5.8
**TS4-n**	3.7	**TS5-n**	3.1	**TS6-n**	3.7
**TS4-x**	4.4	**TS5-x**	3.6	**TS6-x**	4.1
		**TS5-Ch**	10.8		
**25**	−28.3	**26**	−28.3	**27**	27.0
**28**	−27.7	**29**	−27.7	**30**	−27.7
		**31**	−15.3		

**Table 3:** ωB97XD/6-311G(d,p) relative electronic energies in acetonitrile, kcal·mol^−1^, of the stationary points involved in the I-DA reaction of iminium cation **6** with CpMe **20**.

**MC7**	−7.5
**TS7-o**	−0.8
**TS7-m**	2.7
**32**	−24.3
**33**	−24.1

## Data Availability

Data can be made available upon written request to the corresponding author.

## Supplementary Materials

The following are available online. Study of the DA reactions of imines **13** and **14** with Cp **1**. BET study of the I-DA reaction of iminium cation **6** with Cp **1**. Figure with the plot of the asynchronicity vs. the GEDT taking place at eight of the TSs associated with the P-DA and I-DA reactions given in Scheme 1. Figure with the wB97XD/6-311G(d,p) geometries of the exo TSs of the I-DA reactions of iminium cations **6**, **17**–**19** with Cp **1**. Figure with the wB97XD/6-311G(d,p) localization domains and ELF basin attractor positions of the *gauche 60*° **TS2**, the *anti* **TS2-*anti*** involved in the I-DA reaction of Cp **1** with iminium cation **6**, and the point of the IRC from **TS2** in which the C4–C5 distance is similar to that at the *anti* **TS2-*anti***. Table with ωB97XD/6-311G(d,p) electronic energies, in acetonitrile, of the stationary points involved in the I-DA reactions of iminium cations **6**, **15**–**19** with Cp **1**. Table with the ωB97XD/6-311G(d,p) thermodynamic data of the stationary points involved in the I-DA reactions of imine cation **6** with Cp **1**.
