# Supplementary material for: Unveiling the Ionic Diels–Alder Reactions within the Molecular Electron Density Theory
Source: Molecules. 2021 Jun 14;26(12):3638. doi: 10.3390/molecules26123638 (PMC8232178; doi:10.3390/molecules26123638)
Supplement: Supplementary file 1 [file molecules-26-03638-s001.zip › molecules-1255785-supplementary.pdf]

Supplementary Material

Unveiling the Ionic Diels-Alder Reactions within the Molecular Electron Density Theory

Luis R. Domingo,[1*] Mar Ríos-Gutiérrez[1] and María José Aurell[1]

[1]*Department of Organic Chemistry, University of Valencia, Dr. Moliner 50, E-46100 Burjassot, Valencia, Spain,*

E-mail: domingo@utopia.uv.es

Index

S2 Study of the P-DA reactions of imines **13** and **14** with Cp **1**.

S5 BET study of the I-DA reaction of iminium cation **6** with Cp **1**.

S8 Figure with the plot of the asynchronicity *vs* the GEDT taking place at eight of the TSs associated to the P-DA and I-DA reactions give in Scheme 1.

S9 Figure with the ωB97XD/6-311G(d,p) geometries of the *exo* TSs of the I-DA reactions of iminium cations **6**, **17-19** with Cp **1**.

S10 Figure with the ωB97XD/6-311G(d,p) localisation domains and ELF basin attractor positions of the *gauche 60º* **TS2** and the *anti* **TS2-*anti*** involved in the I-DA reaction of Cp **1** with iminium cation **6**, and the point of the IRC from **TS2** in which the C4–C5 distance is similar to that at the *anti* **TS2-*anti***.

S11 Table with ωB97XD/6-311G(d,p) electronic energies, in acetonitrile, of the stationary points involved in the I-DA reactions of iminium cations **6**, **15-19** with Cp **1**.

S12 Table with the ωB97XD/6-311G(d,p)thermodynamic data of the stationary points involved in the I-DA reactions of imine cation **6** with Cp **1**.

1. Study of the P-DA reactions of imines ***13*** *and* ***14*** *with Cp* ***1***

In other to understand the ionic Diels-Alder (I-DA) reactions of iminium cations, the polar Diels-Alder (P-DA) reactions of iminies **13** and **14** with cyclopentadiene (Cp) **1** were also studied. Due to the non-symmetry of imines, the corresponding P-DA reactions can take place through two stereoisomeric reaction paths: the *endo* and the *exo*. These P-DA reactions take place via one-step mechanism. The stationary points involved in the two P-DA reactions are shown in Scheme S1, while the total and relative energies, in gas phase and in acetonitrile, are gives un Table S1.

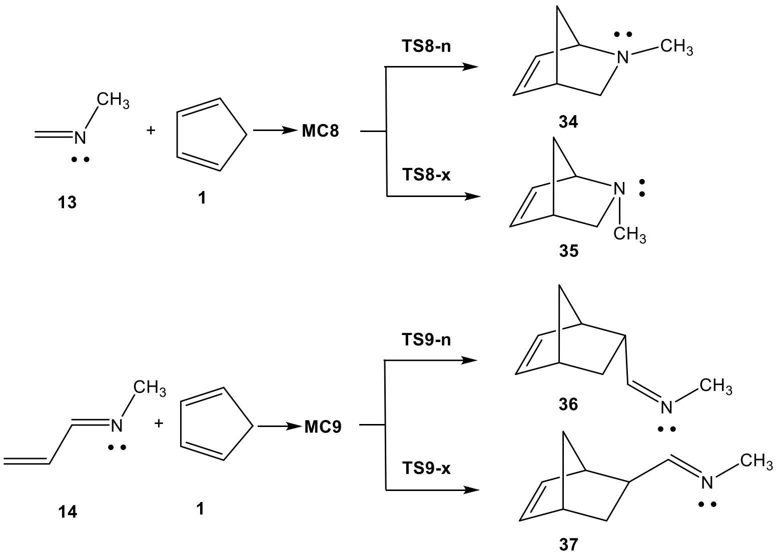

Scheme S1. P-DA reactions of imines **13** and **14** with Cp **1**.

The analysis of the PES associated to these P-DA reactions allowed to find a series of molecular complexes (MCs) in an earlier stage of the reaction in which both reagents are weakly bonded by non-covalent interactions. These MCs are 4.1 (**MC8**) and 4.8 (**MC9**) kcal·mol^{-1} more stable than the separated reagents (see Table S1). The TSs associated to these P-DA reactions are located between 17.3 (**TS9-n**) and 25.7 (**TS8-x**) kcal·mol^{-1} above separated reagents; the reactions being exothermic between 16.6 (**35**) and 26.9 (**36**) kcal·mol^{-1}. Some appealing conclusions can be drawn from the energy results obtained in acetonitrile: i) considering the formation of the MCs, the activation energies associated to these P-DA reactions are 25.7 (**TS8-n**) and 22.1 (**TS9-n**) kcal·mol^{-1}; ii) the P-DA reaction of conjugated imine **14** is kinetically more favourable than the P-DA reaction of imine **13**, in clear agreement with the more electrophilic character of the former (see Table 1); iii) while the P-DA reaction of imine **13** is completely *endo*

selective, the P-DA reaction of conjugated imine **14** is slightly *endo* selective; iv) the P-DA reaction involving conjugate imine **14** , –26.9 (**36**), is more exothermic that the P-DA reaction involving imine **13**, –18.6 (**34**); v) the strong exothermic character of these P-DA reactions, more than 18 kcal·mol^{-1} (**34**), makes them irreversible.

Table S1. ωB97XD /6-311G(d,p) total (E, in a.u) and relative (ΔE, in kcal·mol^{-1}) electronic energies, in gas phase and in acetonitrile, of the stationary points involved in the P-DA reactions of imines **13** and **14** with Cp **1**.

	gas phase		in acetonitrile	
	E	ΔE	E	ΔE
**1**	-194.071440		-194.083783	
**13**	-133.927926		-133.931724	
**MC8**	-328.017362	-11.3	-328.022099	-4.1
**TS8-n**	-327.976641	14.3	-327.981013	21.6
**TS8-x**	-327.969273	18.9	-327.974618	25.7
**34**	-328.042393	-27.0	-328.045139	-18.6
**35**	-328.038377	-24.5	-328.041914	-16.6
**14**	-211.325179		-211.329458	
**MC9**	-405.415374	-11.8	-405.420896	-4.8
**TS9-n**	-405.379435	10.8	-405.385626	17.3
**TS9-x**	-405.378759	11.2	-405.384818	17.8
**36**	-405.451573	-34.5	-405.456037	-26.9
**37**	-405.450915	-34.1	-405.455581	-26.6

The geometries of the TSs are given in Figure S1. The distances between the interacting centers at these TSs in acetonitrile are: 2.030 (C1-C5) and 2.201 (C4-N6) Å at **TS8-n**, 2.018 (C1-C5) and 2.225 (C4-N6) Å at **TS8-x**, 2.083 (C1-C5) and 2.365 (C4-N6) Å at **TS9-n**, and 2.082 (C1-C5) and 2.380 (C4-N6) Å at **TS9-x**. These distances indicate that these TSs are associated to asynchronous processes in which the formation of the C1–C5 single bond is more advanced that the C4–N[C]6 one. In addition, the more favourable *endo* **TS9-n** is slight more delated than the *endo* **TS8-n**.

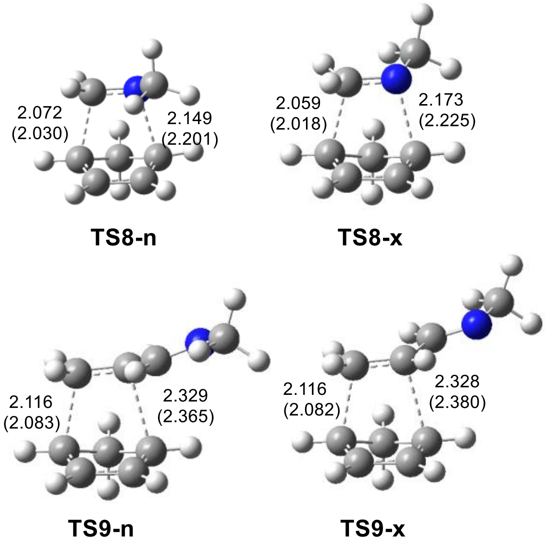

Figure S1. ωB97XD /6-311G(d,p) geometries of the TSs of the P-DA reactions of imines **13** and **14** with Cp **1**. Distances are given in Angstroms. Distance in gas phase are given in parenthesis.

The polar nature of these DA reactions was evaluated by computing the GEDT at the corresponding TSs. Reactions with GEDT values lesser of 0.05 e correspond to non-polar processes, while values higher than 0.2 e correspond to polar processes. The computed GEDT values at the corresponding TSs is 0.12 e at **TS8-n**, 0.14 e at **TS8-x**, 0.09 e at **TS9-n** and 0.09 e **TS9-x**. These values indicate that these DA reactions has some polar character. The TSs associated to the DA reaction involving imine **13** are more polar that those involving imine **14** as the former are more advanced.

*2. BET study of the I-DA reaction of iminium cation **6** with Cp **1**.*

In order to understand the bonding changes along the I-DA reactions and characterize which ones have a greater weight in the corresponding activation energy, a BET study of the I-DA reaction between Cp **1** and iminium cation **6**, was performed. The electron populations of the most relevant valence basins of the selected structures **S1** – **S11** are gathered in Table S2.

According to the BET results presented in Table S2, the mechanism of the I-DA reaction between Cp **1** and iminium cation **6**, can be chemically described as follows:

At the beginning of the reaction, the electronic structure of both frameworks resembles that of the separated reagents. Thus, while the Cp moiety presents a diene structure characterized by two underpopulated C1–C2 and C3–C4 double bonds integrating ca. 3.25 e and one C2–C3 single bond integrating 2.25 e, the iminium moiety presents a C4–N6 double bond with a population of 3.54 e. As the two frameworks start approaching each other, the underpopulated C1–C2 and C3–C4 double bonds of the Cp frameworks are slowly depopulated, at the same time that the C2–C3 region is continuously populated, a behaviour maintained throughout the reaction. On the other hand, the C5–N6 double bond of the iminium starts increasing its population. Note that at this very early stage of the reaction (*Phases I – III*), the GEDT already reaches 0.19 e (see Table S2).

In *Phase IV*, the C5–N6 double bond, which had reached 3.78 e, experiences a significant depopulation to 3.10 e, leading to the N6 non-bonding electron density with an initial population of 0.71 e. Afterwards, the C5–N6 bond keeps being depopulated until the end of the reaction; the released electron density is redistributed either at the C5 carbon and the N6 nitrogen, thus causing the creation of a C5 *pseudoradical* center at **S5** with an initial population of 0.12 e. Similarly, the sudden depopulation of the C1–C2 bonding region of the Cp moiety causes the creation of a C1 *pseudoradical* center integrating 0.31 e at **S6**. Both C1 and C5 *pseudoradical* centers, which are demanded for the subsequent C1–C5 bond formation, reach populations of 0.27 and 0.45 e at the end of *Phase VI*.

When the progress of the reaction reaches **TS2**, the two C1 and C5 *pseudoradical* centers merge each other giving rise to the formation of a notably underpopulated C1C5 single bond integrating 0.76 e. Note that the bond formation is not complete yet.

Then, over a long range of the IRC, the electron density redistribution keeps the same trend; as the C1–C2, C3–C4 and C5–N6 bonding regions become depopulated, the population of the C2–C3 one, and those of the N6 non-bonding electron density and the recently formed C1–C5 bond increases, in such a manner that at the end of *Phase VII*, the C1–C2, C3–C4 and C5–N6 regions can already be considered single bonds, while the C2–C3 region can be considered an underpopulated double bond. On their side, the new C1–C5 single bond reaches 1.72 e and the N6 non-bonding electron density reaches 1.65 e.

At **S8**, a negligible amount of non-bonding electron density integrating 0.06 e appears at the C4 carbon as a consequence of the depopulation of the C3–C4 single bond from 2.44 e at the end of *Phase VII* to 2.38 e. However, this C4 *pseudoradical* center is chemically meaningless because its population increases by only 0.01 e before merging with the N6 non-bonding electron density integrating 1.64 e at the end of *Phase IX* to give rise to the formation of the C4–N6 single bond at **S10** with an initial population of 1.71 e.

Finally, only a reorganization of the electron density to yield the final structure is observed. The only remarkable feature is that the final system ends up with every region being single bonds except one C3–C4 underpopulated double bond.

Table S2. Electron populations of the most relevant ELF valence basins, relative [a] energies, IRC values, GEDT and distances of the forming bonds, for selected structures, **S1** – **S11**, involved in the formation of the C1–C5 and C4–N6 single bonds along the IRC of the I-DA reaction between Cp **1** and iminium cation **6**. Populations and GEDT are given in average number of electrons, e, the ωB97XD /6-311G(d,p) relative energies in kcal·mol^{-1} and distances in angstroms, Å.

Structures	**S1**	**S2**	**S3**	**S4**	**S5**	**S6**	**S7-TS**	**S8**	**S9**	**S10**	**S11**
Phases	*I*	*II*	*III*	*IV*	*V*	*VI*	*VII*	*VIII*	*IX*	*X*	
d(C1-C5)	2.795	2.524	2.400	2.197	2.110	2.081	1.979	1.581	1.579	1.577	1.544
d(C4-N6)	3.286	3.148	3.096	3.016	2.985	2.974	2.936	2.155	2.142	2.114	1.572
IRC	-5.34	-3.19	-2.39	-1.20	-0.72	0.56	0.00	5.50	5.58	5.74	9.41
GEDT	0.09	0.15	0.19	0.29	0.34	0.36	0.43	0.57	0.56	0.56	0.40
ΔE	0.0	1.4	2.6	4.8	5.6	5.8	6.2	-3.6	-3.9	-4.4	-19.4
V(C1,C2)	3.23	3.16	3.12	3.08	3.05	2.74	2.59	2.09	2.08	2.08	1.99
V(C2,C3)	2.25	2.29	2.32	2.39	2.42	2.43	2.49	3.09	1.62	1.69	1.76
V'(C2,C3)									1.49	1.44	1.64
V(C3,C4)	1.76	3.24	3.20	3.12	3.08	3.06	2.99	2.38	2.37	2.35	2.04
V'(C3,C4)	1.54										
V(C5,N6)	1.74	1.78	3.65	3.10	2.92	2.88	2.74	1.88	1.88	1.88	1.82
V'(C5,N6)	1.80	1.83									
V(C1)						0.31					
V(C5)					0.12	0.16					
V(C4)								0.06	0.06		
V(N6)				0.71	0.88	0.94	1.10	1.65	1.65		
V(C1,C5)							0.76	1.73	1.73	1.74	1.86
V(C4,N6)										1.71	1.77

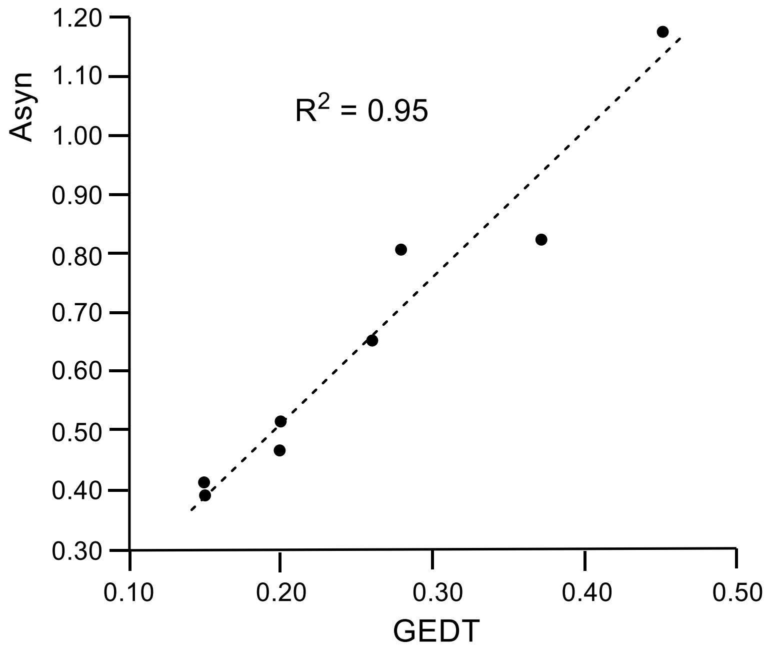

Figure S3. Plot of the asynchronicity, Asyn=(l1-l2) in Angstroms, *vs* the GEDT, in e, taking place at eight of the TSs associated to the P-DA and I-DA reactions give in Scheme 1, R^2 = 0.95.

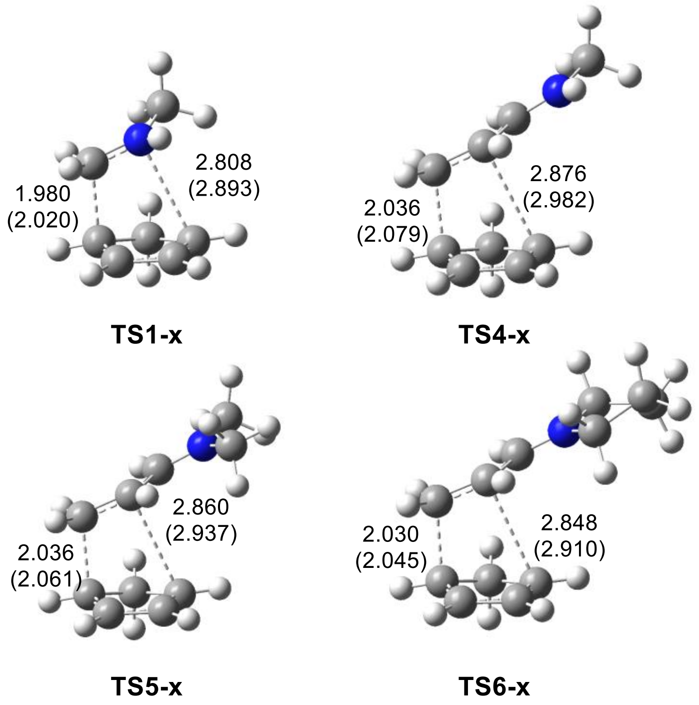

Figure S4. ωB97XD/6-311G(d,p) geometries of the *exo* TSs of the I-DA reactions of iminium cations **6**, **17-19** with Cp **1**. The C−C and C−N distance are given in Angstroms. The gas phase distances are given in parenthesis.

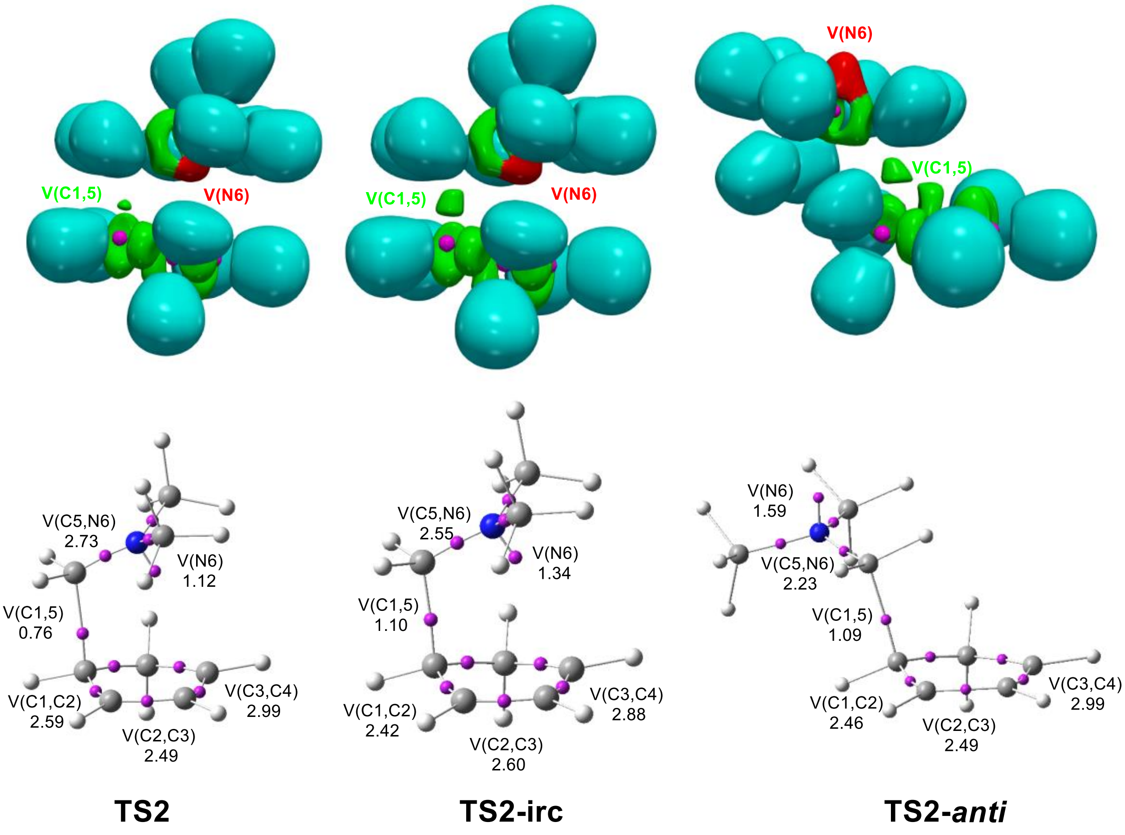

Figure S5. ωB97XD/6-311G(d,p) localisation domains (isosurface value = 0.75), blue colour is used to show protonated basins, red colour for the monosynaptic basins, green colour for the disynaptic basins and magenta colour for core basins, and ELF basin attractor positions, with the most significant valence basin populations, of the *gauche 60º* **TS2** and the *anti* **TS2-*anti*** involved in the I-DA reaction of Cp **1** with iminium cation **6**, and the point of the IRC from **TS2** in which the C4–C5 distance is similar to that at the *anti* **TS2-*anti***.

Table S3. ωB97XD/6-311G(d,p) electronic energies (in a.u.) in acetonitrile of the stationary points involved in the I-DA reactions of iminium cations **6**, **15-19** with Cp **1**.

**1**	-194.083783				
**15**	-134.383434	**6**	-173.693002	**16**	-251.113463
**MC1**	-328.477658	**MC2**	-367.787374	**MC3**	-445.208755
**TS1-n**	-328.468252	**TS2**	-367.772205	**TS3**	-445.192792
**TS1-x**	-328.466194				
**21**	-328.512538	**22**	-367.815098	**23**	-445.236417
**24**	-328.512045				
**17**	-211.786400	**18**	-251.092391	**19**	-328.515380
**MC4**	-405.879161	**MC5**	-445.185165	**TS6-n**	-522.608455
**TS4-n**	-405.864359	**TS5-n**	-445.171237	**TS6-x**	-522.593298
**TS4-x**	-405.863250	**TS5-x**	-445.170390		-522.592620
		**TS5-Ch**	-445.159016	**27**	
**25**	-405.915349	**26**	-445.221218	**30**	-522.644240
**28**	-405.914392	**29**	-445.220301	**TS6-n**	-522.643238
		**31**	-445.200554		

Table S5. ωB97XD/6-311G(d,p) Total enthalpies (H, in a.u.), entropies (S, in cal/mol.K) and Gibbs free energies (G, in a.u.), and relative enthalpies (ΔH, in kcal·mol^{-1}), entropies (ΔS, in cal·mol^{-1}·K) and Gibbs free energies (ΔG, in kcal·mol^{-1}) computed at 25 ºC and 1 atm in acetonitrile, of the stationary points involved in the I-DA reactions of imine cation **6** with Cp **1**.

	H	ΔH	S	ΔS	G	ΔG
**1**	-193.985792		66.6		-194.017454	
**6**	-173.575236		70.5		-173.608755	
**MC2**	-367.569272	-5.2	102.0	-35.2	-367.617728	5.3
**TS2**	-367.554693	4.0	92.1	-45.0	-367.598473	17.4
**22**	-367.592659	-19.8	83.9	-53.3	-367.632505	-4.0
